# Application of Random Mutagenesis and Synthetic FadR Promoter for *de novo* Production of ω-Hydroxy Fatty Acid in *Yarrowia lipolytica*

**DOI:** 10.3389/fbioe.2021.624838

**Published:** 2021-02-22

**Authors:** Beom Gi Park, Junyeob Kim, Eun-Jung Kim, Yechan Kim, Joonwon Kim, Jin Young Kim, Byung-Gee Kim

**Affiliations:** ^1^School of Chemical and Biological Engineering, Seoul National University, Seoul, South Korea; ^2^Institute of Molecular Biology and Genetics, Seoul National University, Seoul, South Korea; ^3^Bio-MAX/N-Bio, Seoul National University, Seoul, South Korea; ^4^Department of Chemical Engineering, Soongsil University, Seoul, South Korea; ^5^Interdisciplinary Program in Bioengineering, Seoul National University, Seoul, South Korea

**Keywords:** *Yarrowia lipolytica*, evolutionary metabolic engineering, synthetic promoter, FadR, ω-hydroxy fatty acid

## Abstract

As a means to develop oleaginous biorefinery, *Yarrowia lipolytica* was utilized to produce ω-hydroxy palmitic acid from glucose using evolutionary metabolic engineering and synthetic FadR promoters for cytochrome P450 (CYP) expression. First, a base strain was constructed to produce free fatty acids (FFAs) from glucose using metabolic engineering strategies. Subsequently, through ethyl methanesulfonate (EMS)-induced random mutagenesis and fluorescence-activated cell sorting (FACS) screening, improved FFA overproducers were screened. Additionally, synthetic promoters containing bacterial FadR binding sequences for CYP expression were designed to respond to the surge of the concentration of FFAs to activate the ω-hydroxylating pathway, resulting in increased transcriptional activity by 14 times from the third day of culture compared to the first day. Then, endogenous *alk5* was screened and expressed using the synthetic FadR promoter in the developed strain for the production of ω-hydroxy palmitic acid. By implementing the synthetic FadR promoter, cell growth and production phases could be efficiently decoupled. Finally, in batch fermentation, we demonstrated *de novo* production of 160 mg/L of ω-hydroxy palmitic acid using FmeN3-TR1-alk5 in nitrogen-limited media. This study presents an excellent example of the production of ω-hydroxy fatty acids using synthetic promoters with bacterial transcriptional regulator (i.e., FadR) binding sequences in oleaginous yeasts.

## Introduction

Construction of biorefinery using microorganisms and biomass can be competitive to that of oil refinery, only when the cost of carbon resource substrates is economical enough to that of petroleum (Cherubini, 2010). Therefore, if the current world petroleum price is persistent at around 35$, the biorefinery using biomass is very difficult to compete with the current petroleum-based economy except for waste carbon resources. Along the same line, biotransformation of fatty acid or fatty acid methyl ester (FAME) hydrolyzed from vegetable oils, mainly palm oil, into bulk chemicals have no price advantage over petroleum-derived fatty acids (Harahap et al., 2019). However, waste vegetable oils and glycerol from food industries are excellent and cheap enough carbon sources to compete with petroleum, since not only the price of the resources but also sustainable society maintenance in terms of carbon recycling give sufficient legitimacy to develop various bioprocesses utilizing the cheap carbon sources (da Silva et al., 2009; Chen et al., 2018). In this regard, unlike *Saccharomyces cerevisiae*, which is characterized to efficiently produce bioethanol from simple sugars such as glucose and sugar cane/beet (Sarkar et al., 2012), non-conventional yeasts have drawn our attention as they have unique traits such as methylotroph or oleaginous yeast strains (Houard et al., 2002; Radecka et al., 2015; Rebello et al., 2018). Among the non-conventional yeasts, *Yarrowia lipolytica* is recognized as a promising industrial strain due to its ability to convert a broad range of carbon substrates such as not only glucose but also glycerol, fructose, fatty acids, and hydrophobic hydrocarbon compounds into high content of intracellular neutral lipids (Barth and Gaillardin, 1996; Mori et al., 2013; Workman et al., 2013; Abghari and Chen, 2014; Lazar et al., 2014) [substrate range of *Y. lipolytica* is well-reviewed herein (Ledesma-Amaro and Nicaud, 2016)]. Primarily, it is notable that waste glycerol from various industries using vegetable oils such as hydrolysis of fats and oil or soap manufacturing could be converted into single-cell oil using *Y. lipolytica* (Dobrowolski et al., 2016). Thus, *Y. lipolytica* is well-suited for the bioproduction of lipid-derived bulk chemicals, as it can assimilate cheap carbon sources, even industrial wastes, into intracellular lipids.

To date, there have been many efforts to utilize *Y. lipolytica* as cell factories for the production of lipid-derived chemicals such as single-cell oil, fatty alcohols, dicarboxylic acids, fatty acid ethyl esters, and medium-chain fatty acids (Gajdoš et al., 2015, 2017; Gao et al., 2018; Mishra et al., 2018; Rigouin et al., 2018; Cordova et al., 2020). Also, since *Y. lipolytica* is notable as a “generally regarded as safe (GRAS)” strain among oleaginous yeasts, it becomes a good cell factory candidate to produce food-grade commercial chemicals such as polyunsaturated fatty acids (Xue et al., 2013; Gemperlein et al., 2019). With these pioneering studies, *Y. lipolytica* showed its potential to be developed as oleo-biorefineries producing a broad range of lipid-derived chemicals, such as detergent, adhesive, dye, lubricant, cosmetics, and polymer and monomer. In parallel with well-developed molecular biology and metabolic engineering tools, now we are entering an era of developing oleaginous biorefinery using *Y. lipolytica*.

The development of synthetic biology, molecular biology tools, and systems biology has remarkably expanded our understanding of non-conventional microorganisms and broadened their applications to design cell factories for industrial biotechnology (Wagner and Alper, 2016; Park et al., 2017). Such new biological and theoretical approaches provided various useful analytical and methodological tools such as metabolic flux analysis, metabolite profiling, promoter engineering, and clusters of regularly interspaced short palindromic repeats (CRISPR)/Cas9 system, enabling us to understand detailed metabolic properties of the microorganisms and industrial host strain development, which are otherwise very tedious and challenging due to dominant non-homologous end-joining (Liu et al., 2016; Weninger et al., 2016; Tredwell et al., 2017; Xiong and Chen, 2020). In particular, non-conventional yeasts, such as *Pichia pastoris*, *Kluyveromyces lactis*, and *Y. lipolytica*, have been recently studied for their potential to implement feasible cell factories for the production of commodity and fine chemicals (Zhu and Jackson, 2015; Wagner and Alper, 2016; Schwarzhans et al., 2017; Abdel-Mawgoud et al., 2018; Cernak et al., 2018).

Recent metabolic engineering and systems biology studies of *Y. lipolytica* revealed underlying mechanisms on how excess carbon sources could be accumulated as neutral lipids in nutrient-limited conditions and how such a metabolic phase shift occurs in *Y. lipolytica* (Beopoulos et al., 2008; Lazar et al., 2018; Wang et al., 2020). In brief, in the absence of nutrients such as nitrogen, intracellular adenosine monophosphate (AMP) decreases as AMP deaminase activity increases. AMP’s low levels inhibit AMP-dependent mitochondrial isocitrate dehydrogenase, accumulating citrate in the mitochondria (Kerkhoven et al., 2016). The accumulated citrate is then transported to the cytoplasm via citrate-malate transporter, and ATP-citrate lyase converts cytoplasmic citrate into oxaloacetate acetyl-CoA. *Y. lipolytica* converts excess carbon sources into cytoplasmic acetyl-CoA throughout the above mechanism and further synthesizes fatty acids and intracellular neutral lipids. If the enriched acetyl-CoA pool is channeled into other valuable chemicals, fatty acid or lipid-derived biorefinery could be easily constructed using the oleaginous yeast as a platform strain.

To construct the biorefinery systems producing fatty acid derivatives, it is essential to design strains that prevent excess carbon sources from being channeled into the synthesis of neutral lipids and to separate cell growth and product production phases since the high expression of heterologous genes for the production of lipid-derived chemicals causes cellular burden to fitness for cell growth. Metabolic engineering of *Y. lipolytica* for the production of free fatty acids (FFAs) has been studied with diverse approaches such as blocking metabolic pathways for synthesis of neutral lipids or degradation of synthesized fatty acids through β-oxidation (Ledesma-Amaro et al., 2016), pushing metabolic flux toward fatty acid synthesis (Ghogare et al., 2020), impairing glycerol metabolism to prevent the synthesis of neutral lipids (Yuzbasheva et al., 2018), expressing heterogeneous thioesterases (Xu et al., 2016), or breaking down lipids to fatty acids and glycerol with overexpression of lipases (Ledesma-Amaro et al., 2016). Also, chimeric eukaryotic type I fatty acid synthase (FAS-I) fused with heterologous thioesterase was applied to release fatty acids directly from acyl-carrier-protein along with genome manipulation using transcription activator-like effector nucleases (TALEN) (Rigouin et al., 2017).

To properly convert the produced fatty acids into target products, it is necessary to induce tailoring enzymes’ expression when fatty acids are accumulated in the cell. In general, according to a desired specific metabolic state, synchronized gene expression is critical to reducing metabolic burdens resulting from the overexpression of the tailoring enzymes. For the synchronization of the enzyme expression, perhaps chemical induction is the easiest method. However, there are drawbacks to finding out inexpensive proper inducers, the need for optimization of induction condition, and low reproducibility because of non-homogeneous mixing of inducers in bioreactors. Moreover, screening inducible promoters responding to the desired cell state is a prerequisite, so that systems biological understanding of the inducible promoters should be preceded. The other alternative method would be using a genetic switch. If a simple genetic switch responding to changes in fatty acid concentrations exists, dynamic control of the tailoring enzymes’ gene expression would be possible for the production of target fatty acid derivatives. Then, cell growth can be easily decoupled with the production phase.

The bacterial FadR is a master regulator involved in lipid biosynthesis and its degradation and regulates the transcription of the related genes by binding to the FadR operator according to the intracellular concentration of fatty acyl-CoA. Studies have been carried out to design genetic circuits or to change the ratio of intracellular saturated or unsaturated fatty acids by redesigning FadR regulon by utilizing characteristics of the bacterial FadR (Teo et al., 2013; Kim et al., 2018). In particular, the previous report investigated the changes in the profile of FadR promoter activity depending on the concentration of externally treated fatty acid, the expression level of FadR, and the number of FadR binding sequences in *S. cerevisiae*. These results showed the possibility of implementing FadR synthetic promoters in *Y. lipolytica* to respond to intracellular fatty acids produced in various nutrient-limited conditions.

ω-Hydroxy fatty acids are valuable chemicals for adhesives, lubricants, and potential anticancer agents (Abe and Sugiyama, 2005; Lu et al., 2010). It can mostly be utilized for renewable monomers for polymers with high resistance to heat and chemicals and biocompatibility (Seo et al., 2015). Also, ω-hydroxylated long-chain fatty acids are essential components of esterified omega-hydroxy ceramides derived from glucosylceramide and sphingomyelin in epithermal cells (LLC, 2017). Since the esterified omega-hydroxy ceramides’ deficiency is strongly correlated with skin diseases such as ichthyosis or atopic dermatitis (Sandhoff, 2010; Breiden and Sandhoff, 2014), the ω-hydroxylated long-chain fatty acids are one of the key components of the skin lipids.

In this study, to develop *Y. lipolytica* as a platform for producing fatty acid derivatives rather than neutral lipids, strains producing FFAs were constructed and improved via random library construction using ethyl methanesulfonate (EMS) mutagenesis and fluorescence-activated cell sorting (FACS) screening, and FadR synthetic promoters were constructed for gene expression responding to the accumulation of fatty acids. To evaluate the developed system, cytochrome P450 (CYP) was used as a model enzyme to convert palmitic acid to ω-hydroxy palmitic acid.

## Materials and Methods

### Strains and Plasmids

All plasmids used in this study were constructed and amplified in *Escherichia coli* DH5α. *Y. lipolytica* Po1g *ku70*Δ strain with weakened homologous recombination was used as the base strain, annotating as wild type (WT) (Verbeke et al., 2013). A list of strains used in this study is summarized in Supplementary Table 1. pCRISPRyl (Addgene #70007) was used to remove genes for strain design as following a protocol from the supplier. N20 sequences of the gene of interest were predicted from “Benchling”^1^ and cloned into pCRISPRyl. Primers used in this study are listed in Supplementary Table 1.

To construct replicating yeast vectors, pCRISPRyl_v1.0 was constructed by deleting the sgRNA expression cassette from pCRISPRyl, and restriction enzymes such as *Sgs*I and *Nhe*I (Thermo Scientific, United States) were used to secure the overexpression vector by replacing the *cas9* gene with the gene of interest.

### Media and Culture Conditions

When culturing *E. coli* DH5α for vector construction and amplification, ampicillin as an antibiotic for selection was added in Luria-Bertani (LB) broth. Yeast Extract-Peptone-Dextrose (YPD) medium containing yeast extract 1% (w/v), peptone 2% (w/v), and glucose 2% (w/v) were used for non-selective seed cultivation of *Y. lipolytica*. For selective cultivation or enrichment of *Y. lipolytica*, synthetic dextrose (SD) media containing glucose 2% (w/v), Yeast Nitrogen Base without amino acids 0.68% (w/v), and 0.05% (w/v) of amino acids and nucleotide mixture without selection marker was used. To induce lipid accumulation in *Y. lipolytica*, CN medium containing glucose 6% (w/v), Yeast Nitrogen Base without amino acids and ammonium sulfate 0.17% (w/v), set amount of ammonium chloride for variation of carbon/nitrogen ratio (CN ratio), and 60 mM phosphate buffer (pH 6.8) was used. If needed, 0.05% (w/v) of amino acids were supplemented to CN medium. For batch fermentation, CN medium having a 60 carbon/nitrogen ratio was used. The batch fermentation’s operation condition proceeded as follows: 1 L working volume, 30°C, 600 rpm, one vvm (vol/vol), and pH was titrated with 3 M KOH to 5.00. All experiments expressing CYP were supplemented with 0.5 mM of 5-aminolevulinic acid and 0.1 mM of ferric sulfate.

### General Molecular Biology Techniques

The transformation of constructed plasmids and relevant editing templates for deleting target genes using the CRISPR/Cas9 system was conducted using a standard lithium acetate protocol (Chen et al., 1997). Briefly, fresh cells from YPD plates and DNAs were mixed with a buffer containing 88 μl of 50% (w/v) PEG 4000, 5 μl of 2 M dithiothreitol, 5 μl of lithium acetate (pH 6.0), and 2 μl of single-strand carrier DNA (10 μg/μl) purchased from Thermo Fisher Scientific. The mixture was incubated at 37°C for 1 h and plated on selective agar plates. To identify positive transformants, 3 μl of 0.02 M sodium hydroxide and a freshly picked colony was boiled at 99°C for 10–15 min. The boiled mixture was then mixed with a PCR reaction mixture containing Taq polymerase (nTaq, Enzynomics).

Gene amplification from genomic DNA was performed by Herculase II Fusion DNA polymerase (Agilent) based on standard recombinant DNA techniques and a supplier’s protocol. For the construction of desired vectors, an amplified DNA and a template vector were cut by restriction enzymes (Thermo Fisher Scientific) and ligated by T4 ligase (Promega). If there were no appropriate restriction enzymes to cut specified DNAs, the circular polymerase extension cloning (CPEC) method was applied (Quan and Tian, 2009).

In detail, promoters with FadR operator sequences were cloned from genomic DNA of *Y. lipolytica* Po1g or in-house vectors. First, GPD promoter (pGPD) was amplified from genomic DNA using pGPD-F and pGPD-front-R, and 17 bp of *E. coli* FadR operator sequence was inserted at the end of pGPD using pGPD-Back-F and pGPD-R, which was fused with overlap extension using primer pGPD-F and pGPD-R. Also, for constructing the same three FadR operator sequences, pGPD was amplified with pGPD-3O-F and pGPD-3O-R, then fused with an amplified fragment using pGPD-BB-F and pGPD-BB-R from pGPD with one FadR operator sequence. For the construction of TEF and LEU promoters harboring FadR operator sequences, each promoter was amplified using primers with the number of operators added. Each fragment was digested with *Pac*I and *Avr*II and ligated to the digested vector harboring pGPD and one or three FadR operator sequences. Humanized *Renilla reniformis* GFP (hrGFP) was amplified from an in-house vector to construct hrGFP-expressing cassette with pGPD using hrGFP-F and hrGFP-R. The digested promoter sequence with *Xba*I and *Eco*RI was ligated with amplified hrGFP digested with *Eco*RI and *Hin*dIII, then subcloned into a pUC19 vector. To construct the yeast-replicating plasmid (YRp) expressing *fadR*, FadR-F and FadR-R were used as primers to amplify *fadR* from an in-house vector. The backbone of YRp was amplified using primers YRp-F and YRp-R from pCRISPRyl v1.0. Then, amplified fragments were ligated using the CPEC method yielding FadR-YRp. Insertion of the hrGFP-expressing cassettes harboring pGPD, pTEF, or pLEU into pFadR-YRp was done by amplifying the hrGFP-expressing cassette from pUC19-hrGFP with hrGFP-YRp-F and hrGFP-YRp-R and ligated with FadR-YRp digested with *Pac*I and *Spe*I.

### Analyses of FFAs and Determination of Lipid Classes

There have been many studies to report analytical methods of FFA quantification. Among them, the following method was performed (Ferreira et al., 2018). Briefly, 5% (v/v) of 40% (w/v) tetrabutylammonium hydroxide was added to the whole broth as a base catalyst, and the same volume of dichloromethane containing 200 mM of methyl iodide was used to convert and extract FFA into FAMEs. The extracted mixture was centrifugated for phase separation. Then, dichloromethane layer was transferred to a new Eppendorf tube for evaporation. Extracted FAMEs were resuspended with hexane and further analyzed in GC/FID (Agilent). Methyl heptadecanoate was used as an internal standard, and analytic standards purchased from Sigma-Aldrich Chemical Co. (St. Louis, MO, United States) were used to obtain standard curves of each FAME. Operation condition for GC/FID was as follows: a non-polar capillary column (5% phenyl methyl siloxane capillary 30 m × 250 m i.d., 0.25 μm film thicknesses) and a linear temperature gradient (60°C 1 min, temperature gradient of 15°C/min to 180°C, hold for 10 min, the temperature gradient of 15°C/min to 200°C, hold for 10 min, the temperature gradient of 15°C/min to 250°C, hold for 10 min) was used.

Lipid classification was performed using thin-layer chromatography (TLC) (Athenstaedt et al., 1999; Schneiter and Daum, 2006). Lipids extracted from lyophilized cells using hexane were applied on silica gel plates, then the plates were developed twice with petroleum ether/diethyl ether/acetic acid [25:25:1 (vol/vol/vol)] and petroleum ether/diethyl ether [49:1 (vol/vol)]. Separated lipids were dyed with iodine vapor for visualization, then additionally charred using the heat gun. Visualized lipids were identified based on the migration of standards purchased from Sigma-Aldrich Chemical Co. (St. Louis, MO, United States).

### Ethyl Methanesulfonate Mutagenesis and Library Screening Using FACS

The random library was constructed via EMS mutagenesis based on a previous study (Liu et al., 2015), and the process was as follows. Overnight seed culture was diluted into 10 OD_600_ units and resuspended in sodium phosphate buffer (pH 7.4). After treating EMS in the buffer to a final concentration of 0.6% (v/v), cells were incubated in a shaking incubator at 30°C for 1 h. Then, the EMS-treated cells were washed with 5% (w/v) sodium thiosulfate, and the cell pellet was resuspended in sterile water. An OD_600_ value of the constructed random library was measured, a small moiety of the sample solution was spread on plates to calculate the size of the random library, and the rest was inoculated into an appropriate medium. All laboratory equipment with contact with EMS were washed thoroughly with 5% (w/v) sodium thiosulfate.

Nile Red was used to stain intracellular lipids, and the following staining method was used: one OD_600_ unit of seed culture was harvested and resuspended in 500 μl of phosphate-buffered saline solution. The resuspended cells were stained with 6 μl of 1 mM Nile Red dissolved in dimethyl sulfoxide and incubated for 15 min in the dark at room temperature. The stained cells were harvested and washed twice with ice-cold sterile water. The stained cells were diluted to 0.1 OD_600_ units for FACS operation (Bio-Rad, S3e Cell Sorter). The FL2 filter was used to determine the fluorescence of stained cells. The filter’s voltage was adjusted to obtain an appropriate chromatogram, and fluorescence was measured under the same conditions for each cycle of iterative enrichment culture and FACS screening. The library was inoculated and cultured in YPD overnight and diluted to a 0.1 OD_600_ unit using the induction medium for lipid accumulation. After the seed culture was fully grown, the cells were harvested and stained with Nile Red. Among the stained cells, the top 5% cells with the highest fluorescence intensity were sorted through FACS machine and inoculated into YPD media for regeneration. The above steps of processes were combined and defined as one cycle (Figure 2A).

### Evaluation of Strength of the Synthetic FadR Promoters via Fluorescence Spectrometry and Quantitative Reverse Transcription-Polymerase Chain Reaction

For evaluation of the strength of synthetic FadR promoters, the fluorescence intensity of hrGFP was measured. The plasmids containing *fadR*-expressing genetic cassette, called pTEF-FadR-Cyc1t, and synthetic FadR promoters expressing hrGFP were transformed into host strains, and transformants were cultured in 2-ml selective media for 24 h at 30°C. The cells were transferred to a 100-ml flask containing 20 ml selective medium. After 12 h of incubation at 30°C, cells were harvested and washed with sterile water. Each strain was diluted to two OD_600_ units in a 4-ml SD medium. Synthetic FadR promoter was induced with exogenously added fatty acid dissolved in dimethyl sulfoxide and Tween-80. After 8 h of incubation at 30°C, OD_600_ was measured using a Labino spectrophotometer (Labotec), and 200 μl of cells were loaded to 96-well microplates (Corning), and their fluorescence was measured with Spark multimode microplate reader (Tecan). Excitation was done with a 485/20 filter, and emission was measured with a monochromator at the wavelength of 520/20.

RNA preparation for quantitative reverse transcription-polymerase chain reaction (qRT-PCR) was conducted using the phenol extraction method (Collart and Oliviero, 2001). Briefly, samples were washed with lysis buffer containing 10 mM Tris–HCl (pH 7.4), 10 mM ethylene diamine tetraacetic acid (EDTA), and 0.5% (w/v) sodium dodecyl sulfate (SDS), and incubated at −70°C for 30 min. After treating with the same amount of acidic phenol, the samples were incubated at 65°C for 20 min. At this time, the samples were vortexed every 5 min. After 10 min of incubation in ice, the samples were centrifuged at 4°C, and supernatant was transferred to new Eppendorf tubes. The same volume of chloroform was added and vortexed. After centrifugation, the supernatant of samples was transferred to new Eppendorf tubes containing 1/10 volume of 3 M sodium acetate and two-volume of 100% ethanol and incubated at −70°C for 30 min. After repetitive washing with 70% ethanol and centrifugation at 4°C, extracted RNA was processed with aspiration and air-drying and eluted with RNase-free water at 65°C.

cDNA was synthesized using M-MLV Reverse Transcriptase (Promega) from the extracted RNA following the supplier’s protocol. For qRT-PCR, TOPreal^TM^ qPCR 2X Premix (Enzynomics, SYBR Green with low ROX) was used as a reaction buffer, and Light Cycler 480 system (Roche) was used to measure relative expression levels of mRNA. To quantify the mRNA level of the gene of interest, *act1* was used as a housekeeping gene, and the temperature gradient was set as follows: preincubation was conducted with a temperature gradient of 4.4°C/min to 95°C and held for 5 min. Amplification was conducted with a temperature gradient of 4.4°C/min to 95°C and held for 20 s, 2.2°C/min to 60°C and held for 20 s, and 4.4°C/min to 72°C and held for 20 s. A total of 40 cycles were repeated for the amplification step. Primers used for qRT-PCR were listed in Supplementary Table 1.

## Results

### Development of *Y. lipolytica* Strains for FFA Production by Iterative Serial Cultures and FACS Screening

**FIGURE 1:**
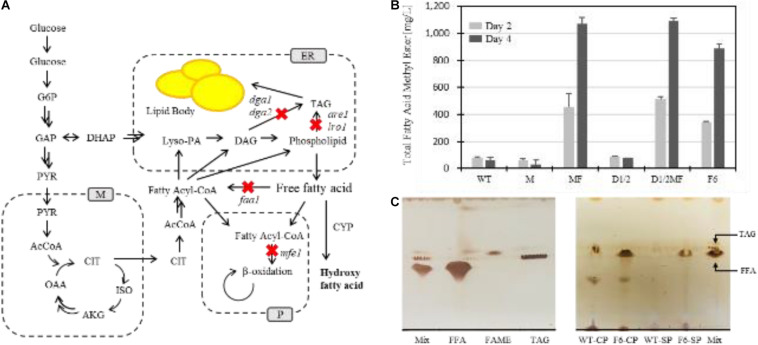
Scheme of metabolic engineering of *Y. lipolytica* for FFA production and evaluation of its production of the constructed strains **(A,B)** and lipid identification using thin-layer chromatography (TLC) analysis **(C)**. Red cross represented deletion of genes involved in marked pathways. Abbreviations used in this figure were as follows: glucose 6-phosphate (G6P), glyceraldehyde 3-phosphate (GAP), dihydroxyacetone phosphate (DHAP), pyruvate (PYR), acetyl-CoA (AcCoA), oxaloacetate (OAA), citrate (CIT), alpha-ketoglutarate (AKG), isocitrate (ISO), lysophosphatidic acid (Lyso-PA), diglyceride (DAG), triglyceride (TAG), mitochondria (M), endoplasmic reticulum (ER), peroxisome (P) free fatty acid (FFA), fatty acid methyl ester (FAME), cell pellet (CP), and supernatant (SP). The strains used in panels **(B)** were as follows: Po1g *ku70*Δ (WT), Po1g *ku70*Δ *mfe1*Δ (M), Po1g *ku70*Δ *mfe1*Δ *faa1*Δ (MF), Po1g *ku70*Δ *dga1*Δ dga2Δ (D1/2), Po1g *ku70*Δ *dga1*Δ dga2Δ *mfe1*Δ *faa1*Δ (D1/2MF), and Po1g *ku70*Δ *dga1*Δ dga2Δ *mfe1*Δ *faa1*Δ *are1*Δ lro*1*Δ (F6). For quantification, total free fatty acids in the strains were converted into total fatty acid methyl esters using the transesterification method described in the “Materials and Methods” section.

Based on pioneering studies on FFA production, metabolic engineering and an evolutionary approach were adopted to improve the FFA production *of Y. lipolytica*. To generate a platform strain to start, a metabolically engineered strain of *Y. lipolytica* was constructed by blocking the biosynthesis of neutral lipids, as the Nicaud group reported (Ledesma-Amaro et al., 2016). To minimize the use of selection markers for further transformation, six genes related to neutral lipids’ biosynthesis were deleted following the previous report (Figure 1A). For rewiring of carbon flux toward FFA production instead of synthesis of neutral lipids, four genes, i.e., two diacylglycerol O-acyltransferases (Dga1 and Dga2), acyl-CoA: sterol acyltransferase (Are1), and acyl-CoA: phospholipid acyltransferase (Lro1) involved in the last step of the biosynthesis of triacylglyceride, were deleted. Also, the two genes involved in activation or degradation of FFA, i.e., acyl-CoA synthetase (Faa1) and peroxisomal multifunctional enzyme (Mfe1), respectively, were deleted for prevention of using FFA-consuming metabolic pathways (Supplementary Figure 1). As a result, based on the constructed strains, i.e., M and MF, hindering FFA consumption, deletion of the genes involved in neutral lipid synthesis were added to MF, yielding D1/2MF and F6.

**FIGURE 2:**
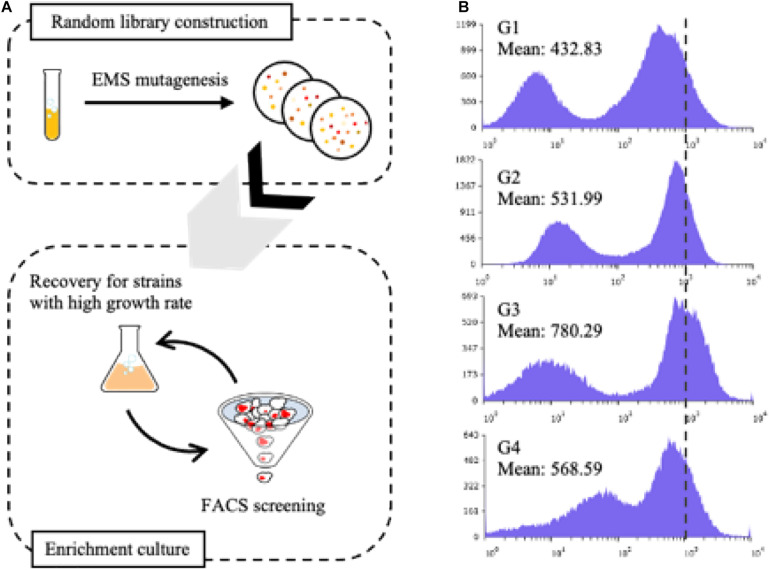
Schematic representation of constructing random library and screening **(A)**. Fluorescence intensity on *X*-axis and cell counts on *Y*-axis were demonstrated in chromatograms acquired from fluorescence-activated cell sorting (FACS) **(B)**. Each cycle of the screening step was annotated as GXs, and the mean value of each cycle’s fluorescence intensity was displayed in the figure. As cycles of screening and enrichment proceeded, G3 showed the highest mean value of fluorescence, and after G3, the value decreased. Thus, further evaluation was conducted using the mutants acquired from G3.

Each mutant’s productivity during the construction of the F6 strain was evaluated in a nitrogen-limited growth medium to induce FFA overproduction (Figure 1B). The F6 strain showed improved productivity about 15 times compared to that of the WT strain. Other strains, such as MF or D1/2MF, also showed improved productivities, about 17 times higher than that of the WT strain. During the construction of the platform strain, one thing to note was that cell growth of the three strains decreased by 20, 30, and 40% compared to that of the WT strain (Supplementary Table 2). Also, to identify lipid classes, TLC analysis revealed that F6 strain accumulated major FFAs in the cytoplasm and minor ones in the supernatant, unlike the WT strain that mainly accumulated neutral lipids in the cell (Figure 1C).

Furthermore, the F6 strain cultured in the nitrogen-deficient medium showed filamentous growth after 100 h (Supplementary Figure 2). As dimorphism of *Y. lipolytica* was reported in the previous studies (Ruiz-Herrera and Sentandreu, 2002), it was hypothesized that filamentous growth could influence growth defect of the F6 strain. To reduce the heterogeneity induced by filamentous growth, Mhy1, i.e., C_2_H_2_-type zinc-finger protein, which is involved in regulating lipid biosynthesis, amino acid and nitrogen metabolism, and cell cycle (Hurtado and Rachubinski, 1999), was additionally deleted, yielding Fm strain. The Fm strain showed unicellular yeast growth and increased its cell growth by 30% (Supplementary Figures 2a,c). Throughout the above metabolic engineering of *Y. lipolytica*, the productivity of FFA in the Fm strain increased significantly (Supplementary Figure 2b). To further overcome the growth defect of the Fm strain, an evolutionary metabolic engineering strategy was attempted. Therefore, it was aimed to screen the strains with recovered cell growth and comparable FFA production. To accelerate the natural evolutionary process for metabolic engineering, a random mutation library of the Fm strain was constructed via EMS mutagenesis. As a result, the experimental group treated with EMS for an hour showed a death rate of 98.9%, and the size of the constructed random library was approximately 8 × 10^6^ cells. A two-step screening method was also designed for screening strains with enhanced growth but with comparable productivity of FFA. It was presumed that iterative serial culture and FACS screening with Nile Red staining could screen robust strains with high yields of FFAs. As the screening cycles proceeded, the mean value of the screened library’s fluorescence intensity increased but started to decrease at the fourth cycle (Figure 2B). Thus, further evaluation was carried out with the strains after the third cycle. After a total of 10 colonies were secured in the screened library, their cell growth rate and fluorescence intensity of the colonies were compared with those of the Fm strain (Table 1). Among them, two strains, annotated as FmeN3 and FmeN4, showed about twofold enhanced fluorescence intensity, and FmeN3 and FmeN6 showed increased cell growth yields by 40 and 90%, respectively. Three candidates (FmeNXs) were selected and further evaluated in the media with different CN ratios.

**TABLE 1:** Colonies from the screened library were compared with the Fm strain.

	Fm 1	Fm 2	N1	N2	N3	N4	N5	N6	N7	N8	N9	N10
OD_600_	1.16	0.78	1.00	1.21	1.38	1.01	1.05	1.81	0.99	1.24	1.05	0.98
Intensity of fluorescence	213.29	208.4	254.03	279.56	419.78	494.86	210.91	244.88	237.32	273.65	305.19	227.75
Fold change compared to mean value of OD_600_			1.03	1.25	1.43	1.04	1.08	1.87	1.02	1.28	1.09	1.01
Fold change compared to mean value of intensity of fluorescence			1.20	1.33	1.99	2.35	1.00	1.16	1.13	1.30	1.45	1.08
Total	1.00		1.24	1.65	2.84	2.45	1.08	2.17	1.15	1.66	1.57	1.09

*OD_600_ and fluorescence-activated cell sorting (FACS) analysis were carried out with samples at 24 h, cultured in the CN 60 media. Mean values acquired from the control strain were used to compare fold changes with growth and fluorescence acquired from 10 colonies. The intensity of fluorescence was measured using FACS. The total change was calculated by multiplying fold changes of OD_600_ and fluorescence intensity. Among 10 colonies, N3, N4, and N6 showed improved growth and fluorescence by about twofold compared to that of the Fm strain.*

**FIGURE 3:**
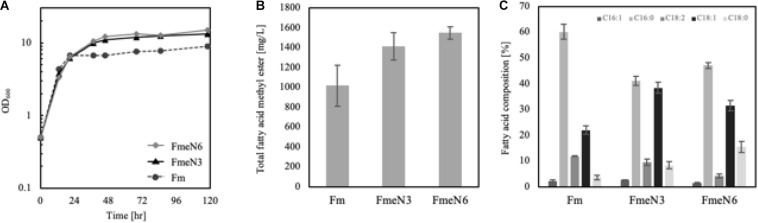
Growth curve **(A)**, free fatty acid (FFA) production **(B)**, and fatty acid composition **(C)** of Fm and the screened mutants cultured in the CN60 medium. Experiments were carried out with biological duplicates. The screened mutants showed better growth and improved FFA production in the nutrient-deficient media than the Fm strain. The composition of fatty acids was calculated by the proportion of each fatty acid over total fatty acid concentration. Each fatty acid was represented in the number of the carbon chain and unsaturated bonds. Error bars represented the standard deviation of biological duplicates.

First, FmeN3, FmeN4, and FmeN6 were cultured in media with a high and low CN ratio, i.e., 120 (C mol/N mol) and 30 (C mol/N mol), respectively, and evaluated for their profiles of cell growth and FFA production under such limiting conditions. It was confirmed that the FmeN3 and FmeN6 showed enhanced cell growth rate and FFA production yields in the medium with low CN ratio, whereas the FmeN4 strain showed lower cell growth rate and FFA production yields under all the conditions examined (Supplementary Figure 3). The experimental results concluded that FmeN4 was false positive. FmeN3 and FmeN6 were further evaluated in the medium with 60 (C mol/N mol) ratio (CN60), inducing the best FFA accumulation when the Fm strain was cultured. After 5 days of culture, the cell growths of FmeN3 and FmeN6 strains were fast by about 50 and 70%, respectively, compared to that of the Fm strain. Also, each strain’s FFA production increased by about 20 and 40%, respectively (Figure 3). In summary, through EMS random mutagenesis and FACS screening method, two strains, FmeN3 and FmeN6, were selected based on the increased cell growth rate and FFA production yields and were further utilized as platform strains for the production of hydroxy fatty acid.

### Construction of FadR Synthetic Promoter Responsive to FFA Production

**FIGURE 4:**
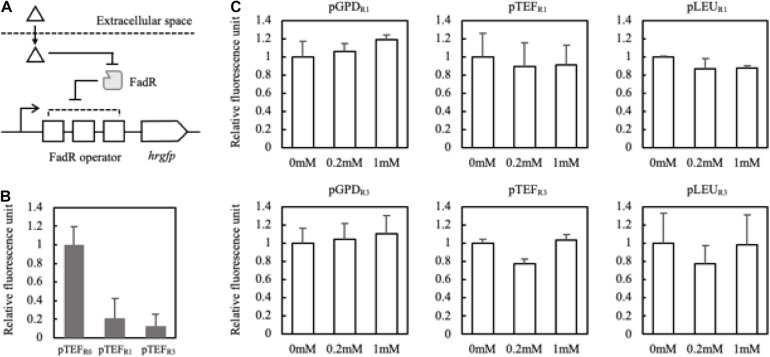
Schematic representation of the FadR synthetic promoters and their induction by extracellularly treated fatty acids **(A)**. Triangle and gray rectangle represented fatty acids and FadR, respectively. The number of FadR binding sequences (R0, R1, and R3) and promoters (pTEF, pGPD, and pLEU) was varied with the construction of the FadR synthetic promoters. Fluorescence intensities of pTEF_R__0_, pTEF_R__1_, and pTEF_R__3_ without extracellularly treated fatty acid were measured **(B)**. Relative fluorescence unit was presented by the concentration of myristic acid on the *X*-axis, which was extracellularly treated, and the synthetic FadR promoters **(C)**. The relative fluorescence unit was calculated based on the synthetic FadR promoters’ fluorescence intensity without inducers (0 mM). Standard deviation was measured in triplicates.

In order to evaluate whether FadR can be used in *Y. lipolytica*, FadR-dependent promoters were constructed and evaluated with an expression of *hrgfp* in FadR-expressing strains. Three well-characterized promoters in *Y. lipolytica*, i.e., glyceraldehyde-3-phosphate dehydrogenase (pGPD), translational elongation factor (pTEF), and 3-isopropyl malate dehydrogenase (pLEU), were used as templates. FadR binding sequences were inserted between the promoters and the start codon. First, FadR-dependent TEF promoters were constructed by harboring varying numbers (i.e., 0, 1, and 3) of FadR binding sequences, yielding pTEF_R__0_, pTET_R__1_, and pTEF_R__3_, respectively. To evaluate the sufficient binding of FadR to its binding sequences, the intrinsic transcription levels of hrGFP of the three promoters were compared in *mfe1* deletion mutant (Figure 4A). pTEF_R__1_ and pTEF_R__3_ showed decreased transcription as fluorescence intensity of hrGFP decreased by 80 and 90%, respectively, compared to pTEF_R__0_ (Figure 4B), suggesting that the transcription of *hrgfp* was inhibited by the constitutively expressed FadR and the presence of the FadR binding sequences. Based on these FadR synthetic promoters, we attempted to confirm whether externally treated fatty acids induced gene expression.

To confirm that externally treated fatty acids can activate the designed synthetic promoters, myristic acid showing a good binding affinity to the FadR was fed with varying concentrations (Teo et al., 2013). pGPD promoters showed about 10% increased fluorescence activity regardless of the copies of binding sequences (Figure 4C). Also, there were no significantly activated promoters among the constructs. The careful examination confirmed that the six FadR promoters’ activity was fully inhibited even in the presence of the added fatty acid, suggesting that externally treated myristic acid did not effectively induce the FadR synthetic promoters. Therefore, to reduce the effect of other factors on FadR promoter strength except FFA concentration, the synthetic promoters’ profile was evaluated in the strains such as Fm endogenously producing FFA *in vivo*.

**FIGURE 5:**
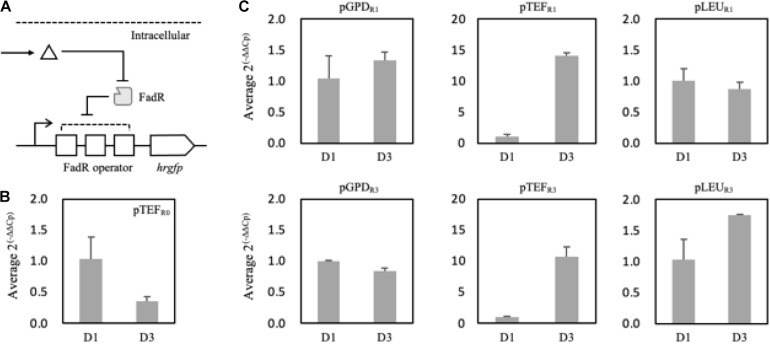
Schematic representation of the FadR synthetic promoters and its induction by intracellularly produced fatty acid **(A)**. Triangle and gray rectangle represented fatty acid and FadR, respectively. The relative mRNA expression level of hrGFP expressed via pTEF without FadR operator (pTEF_R__0_) was compared on days 1 and 3 **(B)**. In panel **(C)**, pGPD, pTEF, and pLEU represented varying promoters constituting the synthetic FadR promoters. R1 and R3 represented the number of FadR operators in the synthetic FadR promoters with varying promoters. mRNAs from each construct were prepared from samples from days 1 and 3. *C*_p_ values were acquired from qRT-PCR, and average 2^(– Δ^ ^Δ^ *^*C*^*^p)^ was calculated using Δ*C*_p_, i.e., *C*_p,__hrGFP_ – *C*_p, Act__1_, of days 1 and 3 of each construct varying promoters and FadR binding sites **(B,C)**. Relative mRNA expression level and standard deviation were measured in triplicate.

To analyze and evaluate the promoters’ transcriptional activity *per se*, quantification of mRNA expression levels of synthetic promoters on the first and third days was carried out as FFA production started from the second day of the culture in the nitrogen-deficient medium. It was revealed that 150 mg/L and 520 mg/L of FFA were produced in the Fm strain on days 1 and 3, respectively (Supplementary Figure 4). On day 3, the relative mRNA expression level of the pTEF_R__0_ was significantly reduced compared to day 1 (Figure 5B). However, pTEF_R__1_ and pTEF_R__3_ displayed about 14 and 11 times higher mRNA expression levels of hrGFP on day 3, respectively (Figure 5C). Even in the cases of pLEU_R__1_ and pLEU_R__3_, the mRNA expression levels decreased by 10% and increased by about 70% on day 3, respectively, compared to day 1 (Figure 5C). The expression profiles of the pGPD_R__1_ and pGPD_R__3_ appeared to be quite different (Figure 5C). For the pGPD_R__1_, the mRNA expression increased by 30% on day 3 compared to day 1. However, the relative mRNA expression level of pGPD_R__3_ decreased by 10% as time passed.

Taken together, the transcriptional activities of the FadR synthetic promoters depend not only on the effector molecule concentrations, i.e., intracellular fatty acid concentrations but also on the strength of promoters in a specific metabolic state. In conclusion, among the various FadR synthetic promoters, pTEF_R__1_ could only decouple cell growth and production of fatty acid derivatives as its transcriptional activity dramatically (i.e., 14 times) increased as FFAs accumulated. Thus, further experiments proceeded with pTEF_R__1_ promoter for the expression of desired enzymes.

### *De novo* Biosynthesis of ω-Hydroxy Palmitic Acid Using FmeN3 Strain Producing FFAs and Expression of *alk5* Under the Control of the FadR Synthetic Promoter

Previously, biotransformation of fatty acids or FAMEs into their corresponding ω-hydroxy fatty acids was carried out using yeasts (Lu et al., 2010; Durairaj et al., 2015) or *E. coli* (van Nuland et al., 2016; He et al., 2019; Yoo et al., 2019) where overexpression of alkane monooxygenase or CYP was attempted to introduce a hydroxy group to the substrates. CYPs drew our attention for their ability to introduce a hydroxy group into many complex structures with high regioselectivity (Urlacher and Girhard, 2019), and their application to the production of fatty acid derivatives in *Y. lipolytica* was quite promising. Firstly, we sought to express several CYPs known as having hydroxylating activity on the omega position of fatty acids in the Fm strain.

**FIGURE 6:**
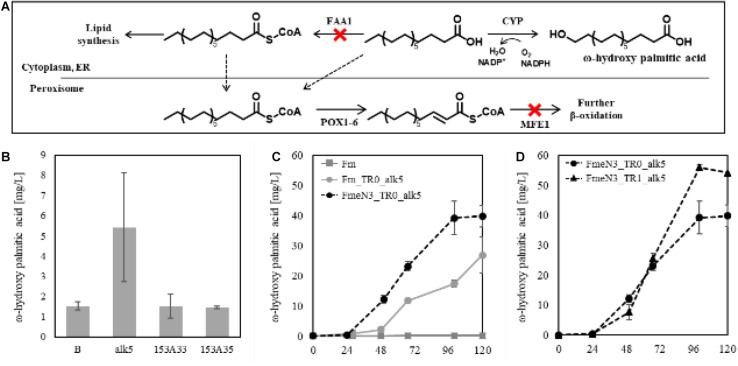
Schematic representation of terminal hydroxylation via CYPs **(A)** and evaluating CYPs in the Fm strain. **(B)** represented the control strain in which the blank vector transformed, and alk5, 153A33, 153A35 represented the experimental strains expressing each CYP in the Fm strain **(B)**. Each CYP was expressed under the UAS1B8-TEF promoter. ω-hydroxy palmitic acid was analyzed using the samples from 2 days after the culture started. Endogenous Alk5 was the most active to palmitic acid produced in the Fm strain. The ω-hydroxy palmitic acid production using the screened mutants and the FadR synthetic promoter was evaluated, respectively **(C,D)**. For the appropriate expression of CYP, 0.5 mM of 5-aminolevulinic acid and 0.1 mM of ferrous sulfate were added when the cultures started. Experiments were carried out with biological duplicates. Error bars represented the standard deviation of biological duplicates. Abbreviations used in panel **(A)** were as follows: Acyl-CoA synthetase (FAA1), cytochrome P450 (CYP), endoplasmic reticulum (ER), Acyl-CoA oxidase (POX), and multifunctional peroxisomal enzyme 1 (MFE1).

According to the Fm platform strains’ profiles of fatty acids, palmitic acid, and oleic acid were the major ones (Figure 3C). To introduce proper CYPs accepting long-chain fatty acids as a substrate into the platform strains, two bacterial CYP153 family members, CYP153A33 and CYP153A35, and one CYP52A family in *Y. lipolytica*, Alk5, were chosen, and they were individually overexpressed in the Fm strain (Scheps et al., 2013; Jung et al., 2018). When cultured in CN60 medium for 2 days, a small amount of ω-hydroxy palmitic acid was produced, and Alk5 showed the highest production yield (5 mg/L) among the three CYPs (Figure 6B). Thus, Alk5 was chosen to further study the production of ω-hydroxy palmitic acid in the screened strains, FmeN3 and FmeN6. Unfortunately, any transformants could not be acquired from FmeN6 for unknown reasons, so that FmeN3 was used as the improved platform strain for the production of ω-hydroxy palmitic acid.

To assess the production of ω-hydroxy palmitic acid using Fm strain and FmeN3 strain containing *alk5* in pTEF_R__0_, yielding Fm_TR0_alk5 and FmeN3_TR0_alk5, respectively, test tube culture was conducted with CN60 medium. After 4 days of cultivation, FmeN3_TR0_alk5 produced about 40 mg/L of ω-hydroxy palmitic acid, which was about a 50% increase in production, compared to Fm_TR0_alk5 (Figure 6C). This result showed that the FmeN3_TR0_alk5 strain is a better performer than the Fm_TR0_alk5 for the production of ω-hydroxy palmitic acid. Next, *alk5* was expressed by pTEF_R__1_ in FmeN3, yielding the FmeN3_TR1_alk5 strain. It was compared with FmeN3_TR0_alk5 to assess its response to FFA accumulated in the nutrient-limited condition, which could decouple cell growth and production of ω-hydroxy palmitic acid (Figure 6D). FmeN3_TR1_alk5 displayed a somewhat (ca. 24 h) delayed product production but resulting in a comparable titer (55 mg/L). Since FFA started to accumulate after the depletion of nitrogen source, in the case of the Fm strain, it took ca. 48 h using the CN60 medium (Figure 1B). Our results also confirmed that the decoupling of cell growth and production of fatty acid derivatives was implemented using FadR synthetic promoters.

### Batch Fermentation

**FIGURE 7:**
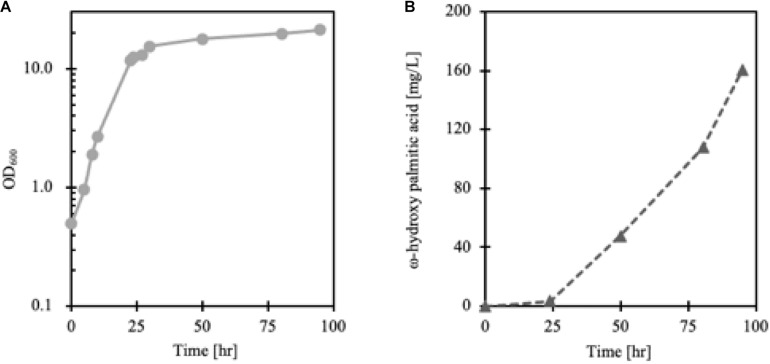
Growth curve **(A)** and production **(B)** of FmeN3_TR1_alk5 cultured in the bioreactor. The dissolved oxygen level of the culture was maintained at over 50% compared to the initial Dissolved Oxygen (DO) level. As cell growth entered the stationary phase after 24 h, ω-hydroxy palmitic acid started to be produced in FmeN3_TR1_alk5.

Lipid accumulation in *Y. lipolytica* was greatly influenced by the degree of aeration so that to synthesize more cellular lipids, non-baffled flask culture was more favorable than baffled flask culture (Xu et al., 2017) (data not shown). However, since the Alk5 reaction requires oxygen as a substrate, to achieve enough oxygen supply for cell growth and maintain a high CYP reaction rate, FmeN3_TR1_alk5 was cultured in a bioreactor. According to the cell growth and FFA production profiles, ω-hydroxy palmitic acid started to be produced in the cell from the stationary phase, around 24 h after the culture started (Figure 7). Here, 160 mg/L of ω-hydroxy palmitic acid, which is about threefold higher titer than that from test tube culture, was yielded at the end of the fermentation run. This result showed that the FadR synthetic promoter strength, the number of FadR binding sites, and oxygen supply were essential parameters to change hydroxy fatty acid production in *Y. lipolytica*.

## Discussion

When *Y. lipolytica* was designed to produce FFA instead of neutral lipid in nutrient-limited conditions, the deletion of acyl-CoA synthetase gene (faa1), involved in modifying fatty acid to fatty acyl-CoA, showed the most significant effect on the production of FFA (Figure 1B). According to some previous studies, there are FAA isozymes in *S. cerevisiae*, and the deletion of multiple faa genes showed increased FFA production (Michinaka et al., 2003; Scharnewski et al., 2008; Chen et al., 2014). We also confirmed that activation of fatty acid synthesis to fatty acyl-CoA and channeling into other pathways consuming fatty acyl-CoA should be avoided to accumulate FFA instead of triacylglyceride (Figure 1C). Also, during our construction of the Fm platform strain, the cell growth rate reduction was somewhat observed as more FFAs were accumulated in the cell.

Previously, EMS was a well-known mutagen for constructing a random library to produce point mutations on the entire microbial genome of interest at a rate of 5 × 10^–4^–5 × 10^–2^ per gene (Wloch et al., 2001; Willensdorfer et al., 2007). According to one of the representative studies from the Alper group, if the mutated *Y. lipolytica* strains accumulate more neutral lipids in the cell, the cells’ specific gravity would decrease and tend to float above liquid media. The floated cells were iteratively screened, and the mutants of enhanced lipogenesis genotype were identified (Liu et al., 2015). To acquire any desired phenotype, it was essential to implement a suitable and optimized screening method. Thus, it was hypothesized that screening fatty acid overproducer with FACS and recovering the cells with higher growth rate among the constructed library should be needed for securing mutants with robust cell growth and comparable accumulation of FFA (Figure 2A). After three generations of iterative FACS screening and EMS mutagenesis, several mutants displaying better cell growth and comparable FFA production were selected (Figure 2B). To identify the mutated genes caused by EMS mutagenesis, the screened mutants will be further subjected to next-generation sequencing (NGS) sequence analysis. Moreover, cell-to-cell variation in all fluorescence results was identified. According to the study, this heterogeneity could be further engineering target to improve FFA production that minimized cell-to-cell variation by implementing a FadR genetic circuit expressing antibiotic transporter in *E. coli* (Xiao et al., 2016).

For implementation of FadR synthetic promoters, FadR protein itself was constitutively expressed under TEF promoter, whereas reporter gene hrGFP was expressed under the modified FadR synthetic promoters in response to the amount of produced fatty acid. We have examined three promoters with different numbers of FadR binding sites and compared their response profiles to the accumulated fatty acid in the Fm strain (Figure 5A). It was confirmed that the response profile significantly changed according to the type of promoter and the number of binding sites constituting the FadR synthetic promoters. We thought this might be influenced by characteristics of the promoter responding to the metabolic status. According to a transcriptomic study on lipid-accumulating conditions in *Y. lipolytica*, there were five clusters categorized by the response profiles of metabolic genes (Morin et al., 2011). Among the study’s identified genes, glyceraldehyde 3-phosphate dehydrogenase (YALI0C06369g, GPD) belonged to cluster 3, which was repressed when lipid started to accumulate. Therefore, the FadR synthetic promoter containing pGPD might be repressed by other factors instead of the FadR/fatty acyl-CoA complex. Also, the dynamic range of FadR synthetic promoters could be controlled by FadR expression level (Teo et al., 2013) and the numbers of FadR binding sites, so that our data showed that various designs would be possible depending upon types and strength of promoter expressing a gene of interest. Also, FmeN3_TR1_alk5 produced a lower amount of ω-hydroxy palmitic acid than FmeN3_TR0_alk5 after 48 h, whereas the maximum productivity of FmeN3_TR1_alk5 increased by 40% than that of FmeN3_TR0_alk5 (Figure 6). The results indicated that as FFA started to be accumulated, the expression of Alk5 by pTEF_R__1_ increased significantly (Figure 5C). After 72 h, the productivity of ω-hydroxy palmitic acid from FmeN3_TR1_alk5 surpassed that of FmeN3_TR0_alk5, where the activity of pTEF_R__0_ would decrease on day 3 (Figure 5B).

Comparing FFA production with or without the introduction of CYPs and the synthetic FadR promoter (Supplementary Figure 5b), Fm_TR0_alk5 and FmeN3_TR0_alk5 showed comparable FFA production with the strains without the introduction of Alk5 (Figure 3B). However, for FmeN3_TR1_alk5, FFA production decreased by 35% compared to that of FmeN3_TR0_alk5. As FadR was constitutively expressed in FmeN3_TR1_alk5, there could be a cellular burden of expressing FadR with the strong promoter, i.e., UAS1B8-pTEF, or FadR might interact with the fatty acid production system in *Y. lipolytica*. In fact, according to fatty acid profiles produced by FmeN3_TR0_alk5 and FmeN3_TR1_alk5 (Supplementary Figure 5c), oleic acid proportion decreased dramatically by about 15% when FadR was expressed in FmeN3_TR1_alk5. FFA production in batch fermentation using FmeN3_TR1_alk5 was about 400 mg/L, which showed lower productivity than FmeN3_TR0_alk5. Based on FFA production results in FmeN3_TR1_alk5, even production of ω-hydroxy palmitic acid increased, FadR expression on FFA production in *Y. lipolytica* needs to be examined in further studies.

This study also showed that FACS analysis and optimization of the bacterial transcriptional regulators as a genetic switch could be preferably utilized to construct oleaginous yeast producing ω-hydroxy fatty acid. Furthermore, this study demonstrated that evolutionary engineering approaches combined with synthetic biology could easily increase oleaginous biorefinery’s potential capacity to higher levels beyond the metabolic engineering approach alone. These approaches can be easily adopted in constructing oleaginous biorefinery producing various chemicals.

## Data Availability Statement

The strains, plasmids, and raw data constructed or generated in this study can be requested from the authors.

## Author Contributions

BP and B-GK conceived the study. BP designed and carried out evolutionary metabolic engineering, FACS screening and application of the synthetic promoters to produce hydroxy fatty acid. BP and E-JK constructed the mutant strains. JuK and BP designed and constructed the synthetic FadR promoters. BP and JuK performed evaluation of the synthetic promoters in *Y. lipolytica*. YK and JYK provided experimental assistance in developing FACS screening and constructing mutants. BP, JoK, and B-GK organized the manuscript. All authors contributed to the article and approved the submitted version.

## Conflict of Interest

The authors declare that the research was conducted in the absence of any commercial or financial relationships that could be construed as a potential conflict of interest.

## Footnotes

1 http://benchling.com

## References

[B1] Abdel-Mawgoud A. M. Markham K. A. Palmer C. M. Liu N. Stephanopoulos G. Alper H. S. (2018). Metabolic engineering in the host *Yarrowia lipolytica*. *Metab. Eng.* 50 192–208. 10.1016/j.ymben.2018.07.016 30056205

[B2] Abe A. Sugiyama K. (2005). Growth inhibition and apoptosis induction of human melanoma cells by omega-hydroxy fatty acids. *Anticancer Drugs* 16 543–549. 10.1097/00001813-200506000-00010 15846120

[B3] Abghari A. Chen S. (2014). *Yarrowia lipolytica* as an oleaginous cell factory platform for production of fatty acid-based biofuel and bioproducts. *Front. Energy Res.* 2:21. 10.3389/fenrg.2014.00021

[B4] Athenstaedt K. Zweytick D. Jandrositz A. Kohlwein S. D. Daum G. (1999). Identification and characterization of major lipid particle proteins of the yeast *Saccharomyces cerevisiae*. *J. Bacteriol.* 181 6441–6448. 10515935 10.1128/jb.181.20.6441-6448.1999 PMC103780

[B5] Barth G. Gaillardin C. (1996). *Yarrowia lipolytica*. *Nonconventional Yeasts in Biotechnology: A Handbook.* Heidelberg: Springer.

[B6] Beopoulos A. Mrozova Z. Thevenieau F. Le Dall M.-T. Hapala I. Papanikolaou S. (2008). Control of lipid accumulation in the yeast *Yarrowia lipolytica*. *Appl. Environ. Microbiol.* 74 7779–7789. 18952867 10.1128/AEM.01412-08 PMC2607157

[B7] Breiden B. Sandhoff K. (2014). The role of sphingolipid metabolism in cutaneous permeability barrier formation. *Biochim. Biophys. Acta* 1841 441–452. 10.1016/j.bbalip.2013.08.010 23954553

[B8] Cernak P. Estrela R. Poddar S. Skerker J. M. Cheng Y. F. Carlson A. K. (2018). Engineering *Kluyveromyces marxianus* as a robust synthetic biology platform host. *mBio* 9 e1410–e1418. e01410-18, 10.1128/mBio.01410-18 PMC6156195 30254120

[B9] Chen C. Sun N. Li D. Long S. Tang X. Xiao G. (2018). Optimization and characterization of biosurfactant production from kitchen waste oil using *Pseudomonas aeruginosa*. *Environ. Sci. Pollut. Res. Int.* 25 14934–14943. 10.1007/s11356-018-1691-1 29549612

[B10] Chen D. C. Beckerich J. M. Gaillardin C. (1997). One-step transformation of the dimorphic yeast *Yarrowia lipolytica*. *Appl. Microbiol. Biotechnol.* 48 232–235. 10.1007/s002530051043 9299782

[B11] Chen L. Zhang J. Lee J. Chen W. N. (2014). Enhancement of free fatty acid production in *Saccharomyces cerevisiae* by control of fatty acyl-coa metabolism. *Appl. Microbiol. Biotechnol.* 98 6739–6750. 10.1007/s00253-014-5758-8 24769906

[B12] Cherubini F. (2010). The biorefinery concept: using biomass instead of oil for producing energy and chemicals. *Energy Convers. Manage.* 51 1412–1421. 10.1016/j.enconman.2010.01.015

[B13] Collart M. A. Oliviero S. (2001). Preparation of yeast RNA. *Curr. Protoc. Mol. Biol.* Chapter 13:Unit13.12. 10.1002/0471142727.mb1312s23 18265096

[B14] Cordova L. T. Butler J. Alper H. S. (2020). Direct production of fatty alcohols from glucose using engineered strains of *Yarrowia lipolytica*. *Metab. Eng. Commun.* 10:E00105. 10.1016/j.mec.2019.e00105 PMC7283507 32547923

[B15] da Silva G. P. Mack M. Contiero J. (2009). Glycerol: a promising and abundant carbon source for industrial microbiology. *Biotechnol. Adv.* 27 30–39. 10.1016/j.biotechadv.2008.07.006 18775486

[B16] Dobrowolski A. Mituła P. Rymowicz W. Mirończuk A. M. (2016). Efficient conversion of crude glycerol from various industrial wastes into single cell oil by yeast *Yarrowia lipolytica*. *Bioresour. Technol.* 207 237–243. 10.1016/j.biortech.2016.02.039 26890799

[B17] Durairaj P. Malla S. Nadarajan S. P. Lee P. G. Jung E. Park H. H. (2015). Fungal cytochrome P450 monooxygenases of *Fusarium oxysporum* for the synthesis of Ω-Hydroxy fatty acids in engineered *Saccharomyces cerevisiae*. *Microb. Cell Fact.* 14 45. 10.1186/s12934-015-0228-2 PMC4387584 25880760

[B18] Ferreira R. Teixeira P. G. Siewers V. Nielsen J. (2018). Redirection of lipid flux toward phospholipids in yeast increases fatty acid turnover and secretion. *Proc. Natl. Acad. Sci. U.S.A.* 115 1262–1267. 10.1073/pnas.1715282115 29358378 PMC5819412

[B19] Gajdoš P. Nicaud J. M. Èertïk M. (2017). Glycerol conversion into a single cell oil by engineered *Yarrowia lipolytica*. *Eng. Life Sci.* 17 325–332. 10.1002/elsc.201600065 32624778 PMC6999298

[B20] Gajdoš P. Nicaud J. M. Rossignol T. Èertïk M. (2015). Single cell oil production on molasses by *Yarrowia lipolytica* strains overexpressing Dga2 in multicopy. *Appl. Microbiol. Biotechnol.* 99 8065–8074. 10.1007/s00253-015-6733-8 26078110

[B21] Gao Q. Cao X. Huang Y. Y. Yang J. L. Chen J. Wei L. J. (2018). Overproduction of fatty acid ethyl esters by the oleaginous yeast *Yarrowia lipolytica* through metabolic engineering and process optimization. *ACS Synth. Biol.* 7 1371–1380. 10.1021/acssynbio.7b00453 29694786

[B22] Gemperlein K. Dietrich D. Kohlstedt M. Zipf G. Bernauer H. S. Wittmann C. (2019). Polyunsaturated fatty acid production by *Yarrowia lipolytica* employing designed myxobacterial pufa synthases. *Nat. Commun.* 10:4055. 10.1038/s41467-019-12025-8 PMC6731297 31492836

[B23] Ghogare R. Chen S. Xiong X. (2020). Metabolic engineering of oleaginous yeast *Yarrowia lipolytica* for overproduction of fatty acids. *Front. Microbiol.* 11:1717. 10.3389/fmicb.2020.01717 32849364 PMC7418586

[B24] Harahap F. Silveira S. Khatiwada D. (2019). Cost competitiveness of palm oil biodiesel production in Indonesia. *Energy* 170 62–72. 10.1016/j.energy.2018.12.115

[B25] He Q. Bennett G. N. San K. Y. Wu H. (2019). Biosynthesis of medium-chain Ω-Hydroxy fatty acids by Alkbgt of *Pseudomonas putida* Gpo1 with native Fadl in engineered *Escherichia coli*. *Front Bioeng Biotechnol* 7:273. 10.3389/fbioe.2019.00273 31681749 PMC6812396

[B26] Houard S. Heinderyckx M. Bollen A. (2002). Engineering of non-conventional yeasts for efficient synthesis of macromolecules: the methylotrophic genera. *Biochimie* 84 1089–1093. 10.1016/s0300-9084(02)00011-1 12595136

[B27] Hurtado C. A. Rachubinski R. A. (1999). Mhy1 encodes a C2h2-type zinc finger protein that promotes dimorphic transition in the yeast *Yarrowia lipolytica*. *J. Bacteriol.* 181 3051–3057. 10.1128/jb.181.10.3051-3057.1999 10322005 PMC93759

[B28] Jung E. Park B. G. Yoo H. W. Kim J. Choi K. Y. Kim B. G. (2018). Semi-rational engineering of Cyp153a35 to enhance Ω-Hydroxylation activity toward palmitic acid. *Appl. Microbiol. Biotechnol.* 102 269–277. 10.1007/s00253-017-8584-y 29124283

[B29] Kerkhoven E. J. Pomraning K. R. Baker S. E. Nielsen J. (2016). Regulation of amino-acid metabolism controls flux to lipid accumulation in *Yarrowia lipolytica*. *NPJ Syst. Biol. Appl.* 2:16005. 10.1038/npjsba.2016.5 PMC5516929 28725468

[B30] Kim J. Yoo H. W. Kim M. Kim E. J. Sung C. Lee P. G. (2018). Rewiring Fadr regulon for the selective production of Ω-Hydroxy palmitic acid from glucose in *Escherichia coli*. *Metab. Eng.* 47 414–422. 10.1016/j.ymben.2018.04.021 29719215

[B31] Lazar Z. Dulermo T. Neuvëglise C. Crutz-Le Coq A. M. Nicaud J. M. (2014). Hexokinase–a limiting factor in lipid production from fructose in *Yarrowia lipolytica*. *Metab. Eng.* 26 89–99. 10.1016/j.ymben.2014.09.008 25307793

[B32] Lazar Z. Liu N. Stephanopoulos G. (2018). Holistic approaches in lipid production by *Yarrowia lipolytica*. *Trends Biotechnol.* 36 1157–1170. 10.1016/j.tibtech.2018.06.007 30006239

[B33] Ledesma-Amaro R. Dulermo R. Niehus X. Nicaud J. M. (2016). Combining metabolic engineering and process optimization to improve production and secretion of fatty acids. *Metab. Eng.* 38 38–46. 10.1016/j.ymben.2016.06.004 27301328

[B34] Ledesma-Amaro R. Nicaud J. M. (2016). Metabolic engineering for expanding the substrate range of *Yarrowia lipolytica*. *Trends Biotechnol.* 34 798–809. 10.1016/j.tibtech.2016.04.010 27207225

[B35] Liu L. Pan A. Spofford C. Zhou N. Alper H. S. (2015). An evolutionary metabolic engineering approach for enhancing lipogenesis in *Yarrowia lipolytica*. *Metab. Eng.* 29 36–45. 10.1016/j.ymben.2015.02.003 25724340

[B36] Liu N. Qiao K. Stephanopoulos G. (2016). 13c metabolic flux analysis of acetate conversion to lipids by *Yarrowia lipolytica*. *Metab. Eng.* 38 86–97. 10.1016/j.ymben.2016.06.006 27387605

[B37] LLC M. (2017). The essential role of esterified omega-hydroxy ceramides as skin lipids. *Newsletter For Glyco/Sphingolipidresearch.* (Matreya Llc, 2nd October 2017).

[B38] Lu W. Ness J. E. Xie W. Zhang X. Minshull J. Gross R. A. (2010). Biosynthesis of monomers for plastics from renewable oils. *J. Am. Chem. Soc.* 132 15451–15455. 10.1021/ja107707v 20936848

[B39] Michinaka Y. Shimauchi T. Aki T. Nakajima T. Kawamoto S. Shigeta S. (2003). Extracellular secretion of free fatty acids by disruption of a fatty acyl-coa synthetase gene in *Saccharomyces cerevisiae*. *J. Biosci. Bioeng.* 95 435–440. 10.1016/s1389-1723(03)80041-5 16233436

[B40] Mishra P. Lee N. R. Lakshmanan M. Kim M. Kim B. G. Lee D. Y. (2018). Genome-scale model-driven strain design for dicarboxylic acid production in *Yarrowia lipolytica*. *BMC Syst Biol* 12(Suppl. 2):12. 10.1186/s12918-018-0542-5 29560822 PMC5861505

[B41] Mori K. Iwama R. Kobayashi S. Horiuchi H. Fukuda R. Ohta A. (2013). Transcriptional repression by glycerol of genes involved in the assimilation of N-Alkanes and fatty acids in yeast *Yarrowia lipolytica*. *FEMS Yeast Res.* 13 233–240. 10.1111/1567-1364.12025 23241327

[B42] Morin N. Cescut J. Beopoulos A. Lelandais G. Le Berre V. Uribelarrea J. L. (2011). Transcriptomic analyses during the transition from biomass production to lipid accumulation in the oleaginous yeast *Yarrowia lipolytica*. *PLoS One* 6:E27966. 10.1371/journal.pone.0027966 22132183 PMC3222671

[B43] Park B. G. Kim M. Kim J. Yoo H. Kim B. G. (2017). Systems biology for understanding and engineering of heterotrophic oleaginous microorganisms. *Biotechnol. J.* 12:1600104. 10.1002/biot.201600104 27809410

[B44] Quan J. Tian J. (2009). Circular polymerase extension cloning of complex gene libraries and pathways. *PLoS One* 4:E6441. 10.1371/journal.pone.0006441 19649325 PMC2713398

[B45] Radecka D. Mukherjee V. Mateo R. Q. Stojiljkovic M. Foulquië-Moreno M. R. Thevelein J. M. (2015). Looking beyond *Saccharomyces*: the potential of non-conventional yeast species for desirable traits in bioethanol fermentation. *FEMS Yeast Res.* 15:fov053. 10.1093/femsyr/fov053 26126524

[B46] Rebello S. Abraham A. Madhavan A. Sindhu R. Binod P. Karthika Bahuleyan A. (2018). Non-conventional yeast cell factories for sustainable bioprocesses. *FEMS Microbiol. Lett.* 365:fny222. 10.1093/femsle/fny222 30212856

[B47] Rigouin C. Croux C. Borsenberger V. Ben Khaled M. Chardot T. Marty A. (2018). Increasing medium chain fatty acids production in *Yarrowia lipolytica* by metabolic engineering. *Microb. Cell Fact.* 17:142. 10.1186/s12934-018-0989-5 PMC6130074 30200978

[B48] Rigouin C. Gueroult M. Croux C. Dubois G. Borsenberger V. Barbe S. (2017). Production of medium chain fatty acids by *Yarrowia lipolytica*: combining molecular design and talen to engineer the fatty acid synthase. *ACS Synth. Biol.* 6 1870–1879. 10.1021/acssynbio.7b00034 28585817

[B49] Ruiz-Herrera J. Sentandreu R. (2002). Different effectors of dimorphism in *Yarrowia lipolytica*. *Arch. Microbiol.* 178 477–483. 10.1007/s00203-002-0478-3 12420169

[B50] Sandhoff R. (2010). Very long chain sphingolipids: tissue expression, function and synthesis. *FEBS Lett.* 584 1907–1913. 10.1016/j.febslet.2009.12.032 20035755

[B51] Sarkar N. Ghosh S. K. Bannerjee S. Aikat K. (2012). Bioethanol production from agricultural wastes: an overview. *Renew. Energy* 37 19–27. 10.1016/j.renene.2011.06.045

[B52] Scharnewski M. Pongdontri P. Mora G. Hoppert M. Fulda M. (2008). Mutants of *Saccharomyces cerevisiae* deficient in acyl-coa synthetases secrete fatty acids due to interrupted fatty acid recycling. *FEBS J.* 275 2765–2778. 10.1111/j.1742-4658.2008.06417.x 18422644

[B53] Scheps D. Honda Malca S. Richter S. M. Marisch K. Nestl B. M. Hauer B. (2013). Synthesis Of Ω-Hydroxy dodecanoic acid based on an engineered Cyp153a fusion construct. *Microb. Biotechnol.* 6 694–707. 10.1111/1751-7915.12073 23941649 PMC3815936

[B54] Schneiter R. Daum G. (2006). Analysis of yeast lipids. *Methods Mol. Biol.* 313 75–84. 16118426 10.1385/1-59259-958-3:075

[B55] Schwarzhans J. P. Luttermann T. Geier M. Kalinowski J. Friehs K. (2017). Towards Systems metabolic engineering in *Pichia pastoris*. *Biotechnol. Adv.* 35 681–710. 10.1016/j.biotechadv.2017.07.009 28760369

[B56] Seo J. H. Lee S. M. Lee J. Park J. B. (2015). Adding value to plant oils and fatty acids: biological transformation of fatty acids into Ω-Hydroxycarboxylic, A, Ω-Dicarboxylic, And Ω-Aminocarboxylic acids. *J. Biotechnol.* 216 158–166. 10.1016/j.jbiotec.2015.10.024 26546054

[B57] Teo W. S. Hee K. S. Chang M. W. (2013). Bacterial fadr and synthetic promoters function as modular fatty acid sensor- regulators in *Saccharomyces cerevisiae*. *Eng. Life Sci.* 13 456–463. 10.1002/elsc.201200113

[B58] Tredwell G. D. Aw R. Edwards-Jones B. Leak D. J. Bundy J. G. (2017). Rapid screening of cellular stress responses in recombinant *Pichia pastoris* strains using metabolite profiling. *J. Ind. Microbiol. Biotechnol.* 44 413–417. 10.1007/s10295-017-1904-5 28160205 PMC5329079

[B59] Urlacher V. B. Girhard M. (2019). Cytochrome P450 monooxygenases in biotechnology and synthetic biology. *Trends Biotechnol.* 37 882–897. 10.1016/j.tibtech.2019.01.001 30739814

[B60] van Nuland Y. M. Eggink G. Weusthuis R. A. (2016). Application of Alkbgt and Alkl from *Pseudomonas putida* Gpo1 for selective Alkyl ester Ω-Oxyfunctionalization in *Escherichia coli*. *Appl. Environ. Microbiol.* 82 3801–3807. 10.1128/aem.00822-16 27084021 PMC4907208

[B61] Verbeke J. Beopoulos A. Nicaud J. M. (2013). Efficient homologous recombination with short length flanking fragments in Ku70 deficient *Yarrowia lipolytica* strains. *Biotechnol. Lett.* 35 571–576. 10.1007/s10529-012-1107-0 23224822

[B62] Wagner J. M. Alper H. S. (2016). Synthetic biology and molecular genetics in non-conventional yeasts: current tools and future advances. *Fungal Genet. Biol.* 89 126–136. 10.1016/j.fgb.2015.12.001 26701310

[B63] Wang J. Ledesma-Amaro R. Wei Y. Ji B. Ji X.-J. (2020). Metabolic engineering for increased lipid accumulation in *Yarrowia lipolytica* – a review. *Bioresour. Technol.* 313:123707. 10.1016/j.biortech.2020.123707 32595069

[B64] Weninger A. Hatzl A.-M. Schmid C. Vogl T. Glieder A. (2016). Combinatorial optimization of Crispr/Cas9 expression enables precision genome engineering in the methylotrophic yeast *Pichia pastoris*. *J. Biotechnol.* 235 139–149. 10.1016/j.jbiotec.2016.03.027 27015975

[B65] Willensdorfer M. Bürger R. Nowak M. A. (2007). Phenotypic mutation rates and the abundance of abnormal proteins in yeast. *PLoS Comput. Biol.* 3:E203. 10.1371/journal.pcbi.0030203 18039025 PMC2082502

[B66] Wloch D. M. Szafraniec K. Borts R. H. Korona R. (2001). Direct estimate of the mutation rate and the distribution of fitness effects in the yeast *Saccharomyces cerevisiae*. *Genetics* 159 441–452. 11606524 10.1093/genetics/159.2.441 PMC1461830

[B67] Workman M. Holt P. Thykaer J. (2013). Comparing cellular performance of *Yarrowia lipolytica* during growth on glucose and glycerol in submerged cultivations. *Amb. Express* 3:58. 10.1186/2191-0855-3-58 24088397 PMC3852309

[B68] Xiao Y. Bowen C. H. Liu D. Zhang F. (2016). Exploiting nongenetic cell-to-cell variation for enhanced biosynthesis. *Nat. Chem. Biol.* 12 339–344. 10.1038/nchembio.2046 26999780

[B69] Xiong X. Chen S. (2020). Expanding toolbox for genes expression of *Yarrowia lipolytica* to include novel inducible, repressible, and hybrid promoters. *ACS Synth. Biol.* 9 2208–2213. 10.1021/acssynbio.0c00243 32584553

[B70] Xu P. Qiao K. Ahn W. S. Stephanopoulos G. (2016). Engineering *Yarrowia lipolytica* as a platform for synthesis of drop-in transportation fuels and oleochemicals. *Proc. Natl. Acad. Sci. U.S.A.* 113 10848–10853. 10.1073/pnas.1607295113 27621436 PMC5047176

[B71] Xu P. Qiao K. Stephanopoulos G. (2017). Engineering oxidative stress defense pathways to build a robust lipid production platform in *Yarrowia lipolytica*. *Biotechnol. Bioeng.* 114 1521–1530. 10.1002/bit.26285 28295166

[B72] Xue Z. Sharpe P. L. Hong S. P. Yadav N. S. Xie D. Short D. R. (2013). Production of omega-3 Eicosapentaenoic acid by metabolic engineering of *Yarrowia lipolytica*. *Nat. Biotechnol.* 31 734–740. 23873085 10.1038/nbt.2622

[B73] Yoo H. W. Kim J. Patil M. D. Park B. G. Joo S. Y. Yun H. (2019). Production of 12-Hydroxy dodecanoic acid methyl ester using a signal peptide sequence-optimized transporter Alkl and a novel monooxygenase. *Bioresour. Technol.* 291:121812. 10.1016/j.biortech.2019.121812 31376668

[B74] Yuzbasheva E. Y. Mostova E. B. Andreeva N. I. Yuzbashev T. V. Fedorov A. S. Konova I. A. (2018). A metabolic engineering strategy for producing free fatty acids by the *Yarrowia lipolytica* yeast based on impairment of glycerol metabolism. *Biotechnol. Bioeng.* 115 433–443. 10.1002/bit.26402 28832949

[B75] Zhu Q. Jackson E. N. (2015). Metabolic engineering of *Yarrowia lipolytica* for industrial applications. *Curr. Opin. Biotechnol.* 36 65–72. 10.1016/j.copbio.2015.08.010 26319895

