# Supplementary material for: Application of Random Mutagenesis and Synthetic FadR Promoter for de novo Production of ω-Hydroxy Fatty Acid in Yarrowia lipolytica
Source: Front Bioeng Biotechnol. 2021 Feb 22;9:624838. doi: 10.3389/fbioe.2021.624838 (PMC7937803; doi:10.3389/fbioe.2021.624838)
Supplement: Supplementary file 1 [file Data_Sheet_1.docx]

Supplementary Material

**Supplementary Table 1.** Strains, vector, and primers used in this study

| Strains used in this study | Organism | Annotation | Strains |  |
| --- | --- | --- | --- | --- |
|  | *E.coli* |  | DH5α | F– φ80lacZΔM15 Δ(lacZYA-argF)U169 recA1 endA1 |
|  |  |  |  | hsdR17(rK–, mK+) phoA supE44 λ– thi-1 gyrA96 relA1 |
| Parent strain | *Y. lipoyltica* |  | Po1g | *MatA, leu2‐270, ura3‐302::URA3, xpr2‐322, axp‐2* |
|  |  | WT | Po1g | *ku70Δ* |
|  |  | M | Po1g | *ku70Δ mfe1Δ* |
|  |  | MF | Po1g | *ku70Δ mfe1Δfaa1Δ* |
|  |  | D1/2 | Po1g | *ku70Δ dga1Δdga2Δ* |
|  |  | D1/2MF | Po1g | *ku70Δ dga1Δdga2Δmfe1Δfaa1Δ* |
|  |  | F6 | Po1g | *ku70Δ dga1Δdga2Δmfe1Δfaa1Δlro1Δare1Δ* |
|  |  | Fm | Po1g | *ku70Δ dga1Δdga2Δmfe1Δfaa1Δlro1Δare1Δmhy1Δ* |
|  |  |  |  |  |
| Sceened mutants from random library | | FmeN3 |  |  |
|  |  | FmeN4 |  |  |
|  |  | FmeN6 |  |  |
|  |  |  |  |  |
|  |  | M_TR0_hrGFP | Po1g | *ku70Δ mfe1Δ* pTEF_R0_-hrgfp pTEF-fadR |
|  |  | M_TR1_hrGFP | Po1g | *ku70Δ mfe1Δ* pTEF_R1_-hrgfp pTEF-fadR |
|  |  | M_TR3_hrGFP | Po1g | *ku70Δ mfe1Δ* pTEF_R3_-hrgfp pTEF-fadR |
|  |  | M_GR1_hrGFP | Po1g | *ku70Δ mfe1Δ* pGPD_R1_-hrgfp pTEF-fadR |
|  |  | M_GR3_hrGFP | Po1g | *ku70Δ mfe1Δ* pGPD_R3_-hrgfp pTEF-fadR |
|  |  | M_LR1_hrGFP | Po1g | *ku70Δ mfe1Δ* pLEU_R1_-hrgfp pTEF-fadR |
|  |  | M_LR3_hrGFP | Po1g | *ku70Δ mfe1Δ* pLEU_R3_-hrgfp pTEF-fadR |
|  |  |  |  |  |
|  |  | Fm_TR0_hrGFP | Po1g | *ku70Δ dga1Δdga2Δmfe1Δfaa1Δlro1Δare1Δmhy1Δ*pTEF_R0_-hrgfp pTEF-fadR |
|  |  | Fm_TR1_hrGFP | Po1g | *ku70Δ dga1Δdga2Δmfe1Δfaa1Δlro1Δare1Δmhy1Δ* pTEF_R1_-hrgfp pTEF-fadR |
|  |  | Fm_TR3_hrGFP | Po1g | *ku70Δ dga1Δdga2Δmfe1Δfaa1Δlro1Δare1Δmhy1Δ* pTEF_R3_-hrgfp pTEF-fadR |
|  |  | Fm_GR1_hrGFP | Po1g | *ku70Δ dga1Δdga2Δmfe1Δfaa1Δlro1Δare1Δmhy1Δ* pGPD_R1_-hrgfp pTEF-fadR |
|  |  | Fm_GR3_hrGFP | Po1g | *ku70Δ dga1Δdga2Δmfe1Δfaa1Δlro1Δare1Δmhy1Δ* pGPD_R3_-hrgfp pTEF-fadR |
|  |  | Fm_LR1_hrGFP | Po1g | *ku70Δ dga1Δdga2Δmfe1Δfaa1Δlro1Δare1Δmhy1Δ* pLEU_R1_-hrgfp pTEF-fadR |
|  |  | Fm_LR3_hrGFP | Po1g | *ku70Δ dga1Δdga2Δmfe1Δfaa1Δlro1Δare1Δmhy1Δ* pLEU_R3_-hrgfp pTEF-fadR |
|  |  |  |  |  |
|  |  | Fm_TR0_A33 |  | *ku70Δ dga1Δdga2Δmfe1Δfaa1Δlro1Δare1Δmhy1Δ* pTEF-cyp153a33 |
|  |  | Fm_TR0_A35 |  | *ku70Δ dga1Δdga2Δmfe1Δfaa1Δlro1Δare1Δmhy1Δ* pTEF-cyp153a35 |
|  |  | Fm_TR0_alk5 |  | *ku70Δ dga1Δdga2Δmfe1Δfaa1Δlro1Δare1Δmhy1Δ* pTEF-alk5 |
|  |  | FmeN3_TR0_alk5 | | FmeN3 pTEF-alk5 |
|  |  | FmeN3_TR1_alk5 | | FmeN3 pTEF_R1_-alk5 |
|  |  |  |  |  |
| Vectors used in this study |  |  |  |  |
|  | Addgene | Specification |  |  |
| pCRISPRyl | #70007 |  |  |  |
| pCRISPRyl_v1.0 |  | template for yeast replicating vector | | |
|  |  |  |  |  |
| pUC19 |  |  |  |  |
| pFadR_yRP |  | FadR cloned to Yrp | |  |
| pUC19_hrGFP |  | hrGFP casette cloned to pUC19 | |  |
| pGPD_R1_ |  | FadR and pGPD_R1_hrGFP cloned to Yrp | | |
| pGPD_R3_ |  | FadR and pGPD_R3_hrGFP cloned to Yrp | | |
| pTEF_R1_ |  | FadR and pTEF_R1_hrGFP cloned to Yrp | | |
| pTEF_R3_ |  | FadR and pTEF_R3_hrGFP cloned to Yrp | | |
| pLEU_R1_ |  | FadR and pLEU_R1_hrGFP cloned to Yrp | | |
| pLEU_R3_ |  | FadR and pLEU_R3_hrGFP cloned to Yrp | | |
|  |  |  |  |  |
| pTEF-alk5 |  | alk5 cloned to Yrp | |  |
| pTEF-cyp153A35 |  | cyp153a35 cloned to Yrp | |  |
| pTEF-cyp153A33 |  | cyp153a33 cloned to Yrp | |  |
| pTEF_R1_-alk5 |  | FadR and pTEF_R1_alk5 cloned to Yrp | | |
|  |  |  |  |  |
| Primiers used in this study |  |  |  |  |
|  |  | Primer |  | Sequence (5' > 3') |
| pCRISPRyl_v1.0 construction | |  |  |  |
|  |  | CRYl_v1_F1_F |  | ATATACTAGTCGTCTCCTCCTCTAGAGTCGAAGCG |
|  |  | CRYl_v1_F1_R |  | ATATCAATTGAAGGGCCAGATCTGTTC |
|  |  | CRYl_v1_F2_F |  | TATACAATTGTCGGTACCCGCTTTTCGTAG |
|  |  | CRYl_v1_F2_R |  | ATATACTAGTCGTCTCCGACTACGTCAGGTGGCACTT |
| FadR plasmids construction |  |  |  |  |
|  |  | FadR_F |  | GAATCATTCAAAGgcgcgccATGGTCATTAAGGCGCAAAGCC |
|  |  | FadR_R |  | TTCTTGGGGTCGGCTCGAGATCGCCCCTGAATGGCTAAATCA |
|  |  | yRP_F |  | TCTCGAGCCGACCCCAAGAA |
|  |  | yRP_R |  | ggcgcgcCTTTGAATGATTC |
|  |  | hrGFP_F |  | TATAGAATTCggCGCGCCATGGTGAGCAAGCAGATCCTGA |
|  |  | hrGFP_R |  | TATAaagcttGCGGCCGCTCGAGCCTCTTCTCTCCAG |
|  |  | pGPD_F |  | TATAtctagaAACCGGACGCAGTAGGATGTCCTGCACGGG |
|  |  | pGPD_Front_R |  | GAAGAAGAAAAAAATCTGGTCGTACCAGATGATGCAGACCCTTATATAAATGTTGCCTTGGACAGACGGAGCA |
|  |  | pGPD_Back_F |  | GTCTGCATCATCTGGTACGACCAGATTTTTTTCTTCTTCTCTTCTCTATATTCATTCTTGAATTAAACAC |
|  |  | pGPD_R |  | TATAGAATTCTGTTGATGTGTGTTTAATTCAAGAA |
|  |  | hrGFP_YRp_F |  | atatTTAATTAAGACGCAGTAGGATGTCCTGC |
|  |  | hrG_YRp_R |  | ATATactagtTCGAGCCTCTTCTCTCCAGG |
|  |  | FadR_BB_F |  | ATATCCTAGGATGGTGAGCAAGCAGATC |
|  |  | FadR_BB_R |  | ATATTTAATTAATTTTCGGGGAAATGTGTG |
|  |  | pGPD_3O_F |  | GTCTGCATCATCTGGTACGACCAGATTTTATCTGGTACGACCAGATTATATTCATTATCTGGTACGACCAGATTCAACAGAAT |
|  |  | pGPD_3O_R |  | GCTCACCATGGCGCGccGAATTCTGTTGAATCTGGTCGTACCAGATAATGAATATAATCTGGTCGTACCAGATAAAATCTGGTCGTACCA |
|  |  | pGPD_BB_F |  | TTCggCGCGCCATGGTGAGCAAGCAGATCCTGAAGAACAC |
|  |  | pGPD_BB_R |  | TCGTACCAGATGATGCAGACCCTTATATAAATGTTGCCTT |
|  |  | TEF_F |  | ATATTTAATTAATTGTGGTTGGGACTTTAG |
|  |  | TEF_1O_R |  | ATATCCTAGGATCTGGTCGTACCAGATTTTGAATGATTCTTATAC |
|  |  | TEF_3O_R |  | ATCCTAGGATCTGGTCGTACCAGATAATGAATATAATCTGGTCGTACCAGATAAAATCTGGTCGTACCAGATTTTGAATGATTCTTATAC |
|  |  | mLeu_F |  | ATATTTAATTAAGCACTGATCACGGGCAAA |
|  |  | mLeu_1O_R |  | ATATCCTAGGATCTGGTCGTACCAGATGTGGATGTGTGTGGTTGT |
|  |  | mLeu_3O_R |  | ATCCTAGGATCTGGTCGTACCAGATAATGAATATAATCTGGTCGTACCAGATAAAATCTGGTCGTACCAGATGTGGATGTGTGTGGTTGT |
| pTEF_R1_ |  |  |  | TTAATTAATTGTGGTTGGGACTTTAGCCAAGGGTATAAAAGACCACCGTCCCCGAATTACCTTTCCTCTTCTTTTCTCTCTCTCCTTGTCAACTCACACCCGAAATCGTTAAGCATTTCCTTCTGAGTATAAGAATCATTCAAAATCTGGTACGACCAGATCCTAGG |
| pTEF_R3_ |  |  |  | ATTAATTAATTGTGGTTGGGACTTTAGCCAAGGGTATAAAAGACCACCGTCCCCGAATTACCTTTCCTCTTCTTTTCTCTCTCTCCTTGTCAACTCACACCCGAAATCGTTAAGCATTTCCTTCTGAGTATAAGAATCATTCAAAATCTGGTACGACCAGATTTTATCTGGTACGACCAGATTATATTCATTATCTGGTACGACCAGATCCTAGG |
| pLEU_R1_ |  |  |  | TTAATTAAGCACTGATCACGGGCAAAAGTGCGTATATATACAAGAGCGTTTGCCAGCCACAGATTTTCACTCCACACACCACATCACACATACAACCACACACATCCACATCTGGTACGACCAGATCCTAGG |
| pLEU_R3_ |  |  |  | TTAATTAAGCACTGATCACGGGCAAAAGTGCGTATATATACAAGAGCGTTTGCCAGCCACAGATTTTCACTCCACACACCACATCACACATACAACCACACACATCCACATCTGGTACGACCAGATTTTATCTGGTACGACCAGATTATATTCATTATCTGGTACGACCAGATCCTAGG |
| pGPD_R1_ |  |  |  | GACGCAGTAGGATGTCCTGCACGGGTCTTTTTGTGGGGTGTGGAGAAAGGGGTGCTTGGAGATGGAAGCCGGTAGAACCGGGCTGCTTGGGGGGATTTGGGGCCGCTGGGCTCCAAAGAGGGGTAGGCATTTCGTTGGGGTTACGTAATTGCGGCATTTGGGTCCTGCGCGCATGTCCCATTGGTCAGAATTAGTCCGGATAGGAGACTTATCAGCCAATCACAGCGCCGGATCCACCTGTAGGTTGGGTTGGGTGGGAGCACCCCTCCACAGAGTAGAGTCAAACAGCAGCAGCAACATGATAGTTGGGGGTGTGCGTGTTAAAGGAAAAAAAAAGAAGCTTGGGTTATATTCCCGCTCTATTTAGAGGTTGCGGGATAGACGCCGACGGAGGGCAATGGCGCCATGGAACCTTGCGGATATCGATACGCCGCGGCGGACTGCGTCCGAACCAGCTCCAGCAGCGTTTTTTCCGGGCCATTGAGCCGACTGCGACCCCGCCAACGTGTCTTGGCCCACGCACTCATGTCATGTTGGTGTTGGGAGGCCACTTTTTAAGTAGCACAAGGCACCTAGCTCGCAGCAAGGTGTCCGAACCAAAGAAGCGGCTGCAGTGGTGCAAACGGGGCGGAAACGGCGGGAAAAAGCCACGGGGGCACGAATTGAGGCACGCCCTCGAATTTGAGACGAGTCACGGCCCCATTCGCCCGCGCAATGGCTCGCCAACGCCCGGTCTTTTGCACCACATCAGGTTACCCCAAGCCAAACCTTTGTGTTAAAAAGCTTAACATATTATACCGAACGTAGGTTTGGGCGGGCTTGCTCCGTCTGTCCAAGGCAACATTTATATAAGGGTCTGCATCATCTGGTACGACCAGATTTTTTTCTTCTTCTCTTCTCTATATTCATTCTTGAATTAAACACACATCAACAGAATTCggCGCGCC |
| pGPD_R3_ |  |  |  | GACGCAGTAGGATGTCCTGCACGGGTCTTTTTGTGGGGTGTGGAGAAAGGGGTGCTTGGAGATGGAAGCCGGTAGAACCGGGCTGCTTGGGGGGATTTGGGGCCGCTGGGCTCCAAAGAGGGGTAGGCATTTCGTTGGGGTTACGTAATTGCGGCATTTGGGTCCTGCGCGCATGTCCCATTGGTCAGAATTAGTCCGGATAGGAGACTTATCAGCCAATCACAGCGCCGGATCCACCTGTAGGTTGGGTTGGGTGGGAGCACCCCTCCACAGAGTAGAGTCAAACAGCAGCAGCAACATGATAGTTGGGGGTGTGCGTGTTAAAGGAAAAAAAAAGAAGCTTGGGTTATATTCCCGCTCTATTTAGAGGTTGCGGGATAGACGCCGACGGAGGGCAATGGCGCCATGGAACCTTGCGGATATCGATACGCCGCGGCGGACTGCGTCCGAACCAGCTCCAGCAGCGTTTTTTCCGGGCCATTGAGCCGACTGCGACCCCGCCAACGTGTCTTGGCCCACGCACTCATGTCATGTTGGTGTTGGGAGGCCACTTTTTAAGTAGCACAAGGCACCTAGCTCGCAGCAAGGTGTCCGAACCAAAGAAGCGGCTGCAGTGGTGCAAACGGGGCGGAAACGGCGGGAAAAAGCCACGGGGGCACGAATTGAGGCACGCCCTCGAATTTGAGACGAGTCACGGCCCCATTCGCCCGCGCAATGGCTCGCCAACGCCCGGTCTTTTGCACCACATCAGGTTACCCCAAGCCAAACCTTTGTGTTAAAAAGCTTAACATATTATACCGAACGTAGGTTTGGGCGGGCTTGCTCCGTCTGTCCAAGGCAACATTTATATAAGGGTCTGCATCATCTGGTACGACCAGATTTTATCTGGTACGACCAGATTATATTCATTATCTGGTACGACCAGATTCAACAGAATTCggCGCGCC |
| Construction of FFA producing strain |  |  |  |  |
|  |  | Mfe1_sgF |  | TGTCAACGATCTTACGTCAACCTGCGCCGA |
|  |  | Mfe1_sgR |  | CCGGGTCGGCGCAGGTTgacGTAAGATCGTTGACAACAACAGGTTTTAGAGCTAGAAATAGC |
|  |  | Mfe1_ET_Up_F |  | CTCCTGAGATCATGCATGCG |
|  |  | Mfe1_ET_Up_R |  | GTGGTGGTCCTAACTGAAACGCAGGTCCGGTATAACTTGA |
|  |  | Mfe1_ET_Dn_F |  | TCAAGTTATACCGGACCTGCGTTTCAGTTAGGACCACCAC |
|  |  | Mfe1_ET_Dn_R |  | CGCTTGAAGAGCCAGCAATC |
|  |  | Faa1_sgF |  | ACgtcAACCTGCGCCGACCC |
|  |  | Faa1_sgR |  | ATGGACGAGGTTGTCGCTCTGTTTTAGAGCTAGAAATAGC |
|  |  | Faa1_ET_Up_F |  | CTGCTCATCTCACTCGTACG |
|  |  | Faa1_ET_Up_R |  | TCTACAATACGCGCTCATCGGATTATGCCGTGGGTTGCTT |
|  |  | Faa1_ET_Dn_F |  | AAGCAACCCACGGCATAATCCGATGAGCGCGTATTGTAGA |
|  |  | Faa1_ET_Dn_R |  | CCACATTTACTACAGCCGAG |
|  |  | Dga1_sgF |  | gacGTtggtggagatgattgctcggGTTTTAGAGCTAGAAATAGC |
|  |  | Dga1_sgR |  | ccgagcaatcatctccaccaACgtcAACCTGCGCCGACCC |
|  |  | Dga1_ET_Up_F |  | atatGGATCCtgtcacctgttgctcacac |
|  |  | Dga1_ET_Up_R |  | gtggagccagcagaTTActccagatccaccgtcttg |
|  |  | Dga1_ET_Dn_F |  | ggtggatctggagTAAtctgctggctccactgcatc |
|  |  | Dga1_ET_Dn_R |  | atatAAGCTTgtcgacagtgtcaaggctg |
|  |  | Dga2_sgF |  | gacGTGAAGAAACCTGCGGGACCCCGTTTTAGAGCTAGAAATAGC |
|  |  | Dga2_sgR |  | ggggtcccgcaggtttcttcACgtcAACCTGCGCCGACCC |
|  |  | Dga2_ET_Up_F |  | ATATGTCGACtacgtgttgcgtttgcacg |
|  |  | Dga2_ET_Up_R |  | aagctggggttggagcacgtcgatttttcgtcgt |
|  |  | Dga2_ET_Dn_F |  | cgaaaaatcgacgtgctccaaccccagcttcaag |
|  |  | Dga2_ET_Dn_R |  | ATATAAGCTTcgacatcatgccgaagaag |
|  |  | Lro1_sgF |  | actcaacgccaagtacccggGTTTTAGAGCTAGAAATAGC |
|  |  | Lro1_sgR |  | AAAACccgggtacttggcgttgagtACgtcAACCTGCGCCGAC |
|  |  | Lro1_ET_Up_F |  | atatGGATCCatctacttgtacgttgtag |
|  |  | Lro1_ET_Up_R |  | catgaagtagaagatgcctgcagaatcttggtcg |
|  |  | Lro1_ET_Dn_F |  | ccaagattctgcaggcatcttctacttcatgaag |
|  |  | Lro1_ET_Dn_R |  | atatAAGCTTcgtcgttcgtcgttcgtcg |
|  |  | Are1_sgF |  | gctcaatgtgcccgacggccGTTTTAGAGCTAGAAATAGC |
|  |  | Are1_sgR |  | AAAACggccgtcgggcacattgagcACgtcAACCTGCGCCGAC |
|  |  | Are1_ET_Up_F |  | atatGGATCcgtactgtactatgaaacac |
|  |  | Are1_ET_UP_R |  | atatCTCGAGTTAcattgtgtgtgcggagagtg |
|  |  | Are1_ET_Dn_F |  | ATATCTCGAGcaccgacatcaagttcaag |
|  |  | Are1_ET_Dn_R |  | AATTGAATTCgcaaacatgaccagctcg |
|  |  | Mhy1_sgF |  | gacGTGGAGATGGCGAGATAACGGAGTTTTAGAGCTAGAAATAGC |
|  |  | Mhy1_sgR |  | TCCGTTATCTCGCCATCTCCACgtcAACCTGCGCCGACCC |
|  |  | Mhy1_ET_UP_F |  | ATATGAATTCCCAACAGATGGCCCATAC |
|  |  | Mhy1_ET_UP_R |  | ATATGGATCCCGAGGTCCATTTTGGCG |
|  |  | Mhy1_ET_Dn_F |  | ATATGGATCCGTTGAAGAGCGAGCCCA |
|  |  | Mhy1_ET_Dn_R |  | ATATGTCGACGGCGAGTACGAAACCTC |
|  |  |  |  |  |
|  |  | Alk5_XmaJI_TEF_F |  | ATATCCTAGGGCAATATGCTACAACTCTTTGGCG |
|  |  | Alk5_SpeI_TEF_R |  | ATATACTAGTCGACGGAATTCTCATGT |
|  |  | 153A33_F_CPEC |  | tcattcaaaggcgcgccacaATGCCAACACTGCCCAG |
|  |  | 153A33_R_CPEC |  | actaattacatgaggctagcTTAACTGTTCGGTGTCAGT |
|  |  | 153A35_F_AscI |  | ATATggcgcgccATGCAGATCCTCGACCG |
|  |  | 153A35_R_NheI |  | ATATgctagcTCATGACCGTGTCTTCGGC |
|  |  | Alk5_F_AscI |  | ATATggcgcgccATGCTACAACTCTTTGGCG |
|  |  | Alk5_R_NheI |  | ATATgctagcCTACGCCTTCTCACCCTTA |

|  | **OD_600, f_** | **FFA titer**  **[mg/L]** | **Specific FFA titer**  **[mg/L/OD unit]** | **Yield**  **[g FFA/g glucose]** |
| --- | --- | --- | --- | --- |
| **WT** | 22.9 ± 0.9 | 62.5 ± 20.1 | 2.8 ± 1.0 | 0.002 ± 0.001 |
| **M** | 21.1 ± 0.4 | 30.6 | 1.5 | 0.001 |
| **MF** | 17.8 ± 0.4 | 1068.0 ± 47.8 | 60.0 ± 1.3 | 0.036 ± 0.004 |
| **D1/2** | 17.5 ± 0.8 | 78.2 ± 0.6 | 4.5 ± 0.2 | 0.003 ± 0.001 |
| **D1/2MF** | 15.6 ± 1.0 | 1094.2 ± 18.0 | 70.5 ± 3.3 | 0.043 ± 0.007 |
| **F6** | 13.3 ± 0.3 | 888.3 ± 31.8 | 66.7 ± 3.8 | **0.056 ± 0.007** |

## Supplementary Table 2.

Culture parameters of the constructed mutants in the nitrogen-limited media after four days. Consumed glucose was calculated from residual glucose analyzed by LC. Duplicate experiments were performed with collected samples. Compared to WT, the F6 strain showed impaired growth and significantly improved production yield. The shaded row represented the constructed mutants that produced a noticeable amount of FFA. During the construction of deletion mutants, deletion of *faa1* showed a significant effect on FFA production.


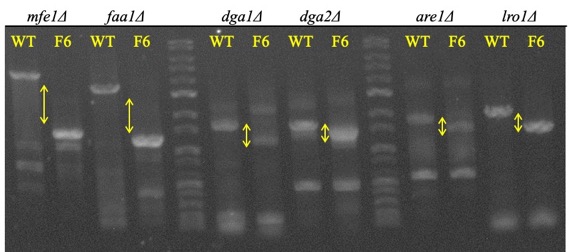


Supplementary Figure 1. Confirmation of deletion of targeted genes in F6 strain compared to WT by PCR amplification. The yellow arrow represented the deleted region in the gene of interest. CRISPR/Cas9 system was applied to delete target genes, and sgRNAs were designed to bind to the nearby start codon. After a double-strand break induced by Cas9 protein, homologous recombination was proceeded using editing template harboring stop codon.


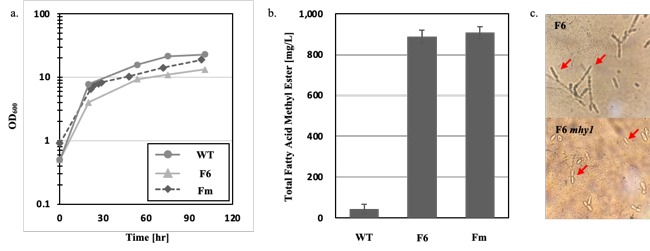


Supplementary Figure 2. Growth curve (a), FFA production (b), and microscopic examination (c) of F6 and Fm strain when the mutants cultured in nitrogen-limited conditions. Microscopic examination was carried out with the samples at 100hrs using Olympus BX51. Red arrow showed that filamentous growth of F6 strain and unicellular yeast growth of Fm strain. Experiments were carried out with biological duplicates. Error bars represented the standard deviation of biological duplicates.


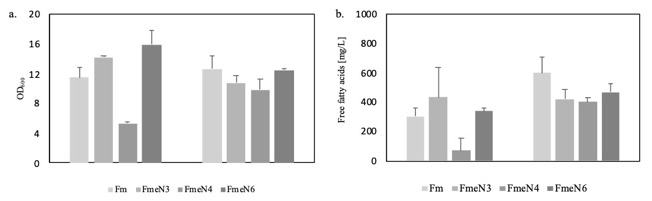


Supplementary Figure 3. OD_600_ (a) and FFA production (b) of Fm and screened mutants in CN ratio of 30 (low) and 120 (high) were analyzed at 100 hrs. The experiments were performed with biological duplicates. FmeN3 and FmeN6 showed better cell growth and FFA production in CN30 media, however, Fm strain showed better performance in CN120 media. FmeN4 showed impaired growth and FFA production compared to Fm strain and was excluded for further evaluation.


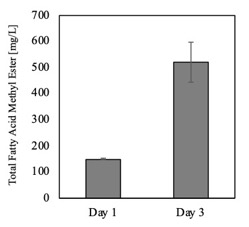


Supplementary Figure 4. Free fatty acid production in the Fm strain when mRNA was prepared for evaluating the synthetic FadR promoters on day 1 and day 3. While production of free fatty acid increased, the relative expression level of mRNA of some synthetic FadR promoters, i.e., pTEF_R1_, pTEF_R3_, and pLEU_R3_, increased as in Figure 5. The experiments were performed using biological duplicates, and the error bar represented the standard deviation.


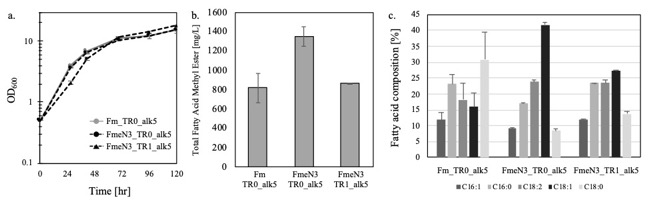


Supplementary Figure 5. Growth curves(a), FFA production (b), and profile (c) of Fm and F3 expressing alk5 with or without the synthetic FadR promoter. The experiments were performed with biological duplicates. Cell growth was not significantly different among the three strains, but the fatty acid profile differed between the Fm strain and the F3 strains. The Fm strain had a high proportion of saturated fatty acids, while the F3 strains had the highest proportion of oleic acid. Error bars represented the standard deviation of biological duplicates.
